## [Peer Review File · Nature Communications]

Reviewers' Comments:

Reviewer #1:

Remarks to the Author:

Nielsen and co-workers present a new version of GECKO, Genome-scale metabolic models with Enzyme Constraints using Kinetic and Omics data. With respect to their previous work, they aim to automate the generation of enzyme-constrained models and identification of kinetic parameters for less studied organisms. It is remarkable the effort in the visualization capabilities and complementary analyses associated with GECKO, as well as tools necessary for community development. However, I find several issues that need to be addressed and question the major contribution of this new version of GECKO.

1. Authors mention in Lines 151-152 that the results derived from GECKO "are highly sensitive to the distribution of incorporated kinetic parameters". However, they do not sufficiently describe how they address this issue. We can find the same enzyme with very different *k_{cat}* under different experimental contexts. How general are the values provided in BRENDA for the simulated conditions? In fact, authors show in Supplementary Note 1 the dispersion found in different enzymes. However, they do not explain which *k_{cat}* value must be taken. For example, the median value, maximum value?
2. "A modified set of hierarchical *k_{cat}* matching criteria was implemented as part of GECKO 2.0" (Lines 175-176). This is possibly the most important change with respect to the previous version. It should be described in Methods section and not in Supplementary Note 2. For this reason, Figure 1D is difficult to follow in the main text. How did authors validate the use of a phylogenetic distance to infer *k_{cat}*s?
3. Authors report an improvement in predicting batch growth in 19 environments with respect to their previous algorithm: 23.97% vs 32.07 relative error? Is that difference significant? How was this error calculated? Did authors calibrate the model using sensitivity analysis? This is something tricky and overfitting could be a problem in the derived model? Did authors test their new model with experimental data that were not used to calibrate the model? This is important and not described in the text.
4. Figure 3: Authors report an improvement in growth rate prediction capacity of *E. coli* in comparison with ME-model. Could authors show correlation instead of sum of square errors (SSE)?
5. Figure 4: "Additionally, the incorporation of individual protein abundance constraints improves even further the predictive accuracy of gaseous exchanges, for 10 out of the 11 evaluated cases". I am missing something. I can see 4 points out of 11 where no improvement is obtained: std in yeast, std *y_{osm}* in *K. marxianus* and HiT in *Y. lipolytica*. Is this result statistically significant? Has this experimental design enough power? Given the number of parameters required in ec-Models, in comparison with GEMs, could it be this only overfitting?

Minor comments

What do authors mean by "wild-cards"? (Line 189)

Reviewer #2:

Remarks to the Author:

Domenzain et al., have presented the updated version of GECKO toolbox in this paper and have demonstrated creation of high-quality enzyme constrained models (ecModels) for functional GEMs. The work is of relevance to the constraint-based modeling field. The paper is well-written. The following are some comments related to the work presented in this paper:

1. The authors mention that additional condition-dependent exchange fluxes of byproducts can be used as constraints in order to limit the feasible solution space. How does the user decide which condition-specific exchange fluxes need to be used in a particular condition? Can the authors support this with an example?

2. Can the authors define what are "active fluxes" in the context of metabolic fluxes?
3. How does the method handle reactions that are not associated with enzymatic activities? For example, some exchange and transport reactions that do not have any GPRs associated.
4. Based on the Kcat distribution in BRENDA, is it possible to predict the minimum cut-off needed for constraining the GEM of other organisms like Mtb, Mus musculus, Arabidopsis etc.?
5. In case of multi-omics data available for organisms like yeast and humans, is it possible to integrate that information in the model along with the proteins to constrain the model and obtain biologically meaningful predictions?
6. How much is the computing time needed for analyzing the ecModels considering the fact that they are of different sizes (reactions, metabolites and proteins)?

Minor comments:

1. The term "eukaryal" is not commonly used term. It is more appropriate to use the term "eukaryote" for *S. cerevisiae*.
2. In line 363-364 the authors mentioned "This suggests that upper bounds on enzyme usages induce differentiated utilization of isoenzymes, reflecting well why isoenzymes have been maintained throughout evolution". Can the authors elaborate more on some examples of isozymes to support their point?
3. Did the authors use fastFVA for the analysis shown in this paper?

Revision cover letter

with detailed point-by-point responses to the reviewers' comments

Reviewer #1 (Expertise: Metabolic modelling):

Nielsen and co-workers present a new version of GECKO, Genome-scale metabolic models with Enzyme Constraints using Kinetic and Omics data. With respect to their previous work, they aim to automate the generation of enzyme-constrained models and identification of kinetic parameters for less studied organisms. It is remarkable the effort in the visualization capabilities and complementary analyses associated with GECKO, as well as tools necessary for community development. However, I find several issues that need to be addressed and question the major contribution of this new version of GECKO.

R: We thank the reviewer for the feedback and the kind words. Since it is not part of the subsequent remarks, we would like to mention here that the last paragraph of the *Introduction* has been rephrased to better capture the advances in GECKO 2.0. The original paragraph was split into two, and the original "Here we wanted to build GECKO models for several organisms, and we therefore updated the GECKO toolbox to its 2.0 version." was changed to "In this work, we updated the GECKO toolbox to its 2.0 version, expanding its use it for building enzyme-constrained models (ecModels) for more organisms."

1. Authors mention in Lines 151-152 that the results derived from GECKO "are highly sensitive to the distribution of incorporated kinetic parameters". However, they do not sufficiently describe how they address this issue. We can find the same enzyme with very different k_{cat} s under different experimental contexts. How general are the values provided in BRENDA for the simulated conditions? In fact, authors show in Supplementary Note 1 the dispersion found in different enzymes. However, they do not explain which k_{cat} value must be taken. For example, the median value, maximum value?

R: We thank the reviewer for the remark.

The original line 148 has been revised to clarify that the previously published GECKO 1.0 was observed to be sensitive to the distribution of k_{cat} values by changing "The first implementation of the GECKO method" to "The previous implementation of the GECKO method in GECKO 1.0".

In addition, lines 175-176 were revised to clarify that in GECKO 2.0 the hierarchical k_{cat} matching algorithm is aimed at improving this distribution, changing "As k_{cat} numbers depend on biochemical mechanisms, metabolic context and phylogeny of host organisms, a modified set of hierarchical k_{cat} matching criteria was implemented as part of GECKO 2.0" to "In the new version of the GECKO toolbox (GECKO 2.0), a modified set of hierarchical k_{cat} matching criteria was implemented to address how k_{cat} numbers depend on biochemical mechanisms, metabolic context and phylogeny of host organisms."

Moreover, lines 182-183 have been revised to also mention that the algorithm is fully detailed in Supplementary File 2 by changing "A comparison of the new k_{cat} matching criteria with their predecessor set is shown in Supp. file 2" to "The new k_{cat} matching algorithm, including the estimation of the phylogenetic distance, and its comparison with the predecessor are shown in Supplementary File 2".

2. "A modified set of hierarchical k_{cat} matching criteria was implemented as part of GECKO 2.0" (Lines 175-176). This is possibly the most important change with respect to the previous version. It should be described in Methods section and not in Supplementary Note 2. For this reason, Figure 1D is difficult to follow in the main text. How did authors validate the use of a phylogenetic distance to infer k_{cat} s?

R: We thank the reviewer for identifying an important piece of our work. A new sentence was introduced to summarize this change in k_{cat} matching: “Specifically, in cases where GECKO 1.0 would have chosen available k_{cat} values for any organism, GECKO 2.0 chooses those available for the phylogenetically closest ones by iteratively introducing a wildcard into the E.C. number, as exemplified in **Supplementary File 1** “EC3.x.x.x”, and estimating the phylogenetic distance.”

This newly added line is also aimed to clarify that k_{cat} values are merely sorted by, and not inferred from, the phylogenetic distance. In order to explain this change in full length while respecting editorial formatting constraints, the sections detailing it, including Table S2.1., have been kept in Suppl. file 2.

Moreover, the main text mentioning Fig. 1D has also been revised, from “A classification of the matched k_{cat} numbers according to the new matching algorithm is provided in Fig. 1D “ to “A classification of the matched k_{cat} values according to the new matching algorithm is provided in Fig. 1D, showing the amount of values chosen from the phylogenetically closest organisms”.

3. Authors report an improvement in predicting batch growth in 19 environments with respect their previous algorithm: 23.97% vs 32.07 relative error? Is that difference significant? How was this error calculated? Did authors calibrate the model using sensitivity analysis? This is something tricky and overfitting could be a problem in the derived model? Did authors test their new model with experimental that were not used to calibrate the model? This is important and not described in the text.

R: We thank the reviewer for the interest in the comparison between GECKO 2.0 and GECKO 1.0 via ecYeast7. In the spirit of reproducibility, as mentioned in the Code Availability section, all custom scripts for simulations included in this study can be found at https://github.com/SysBioChalmers/GECKO2_simulations/, including Figure 1G which is generated by running the MATLAB script *Csources_simulations.m*, available at: code/GECKO_versions_comparison. The script also includes the calculation of the relative error for the simulation in the variable ‘res’.

4. Figure 3: Authors report an improvement in growth rate prediction capacity of E. coli in comparison with ME-model. Could authors show correlation instead of sum of square errors (SSE)?

R: We thank the reviewer for suggesting this improvement. The description of Figure 3 has been revised accordingly by adding “The R^2 values are 0.196 for ec/ML1515, 0.285 for ME-/JL12678 optimal and 0.178 for ME-/JL12678 generalist”.

5. Figure 4: “Additionally, the incorporation of individual protein abundance constraints improves even further the predictive accuracy of gaseous exchanges, for 10 out of the 11 evaluated cases”. I am missing something. I can see 4 points out of 11 where no improvement is obtained: std in yeast, std y osm in K. marxianus and HiT in Y. lypolytica. Is this result statistically significant? Has this experimental design enough power? Given the number of parameters required in ec-Models, in comparison with GEMs, could it be this only overfitting?

R: We are very thankful to the reviewer for closely observing the figures and noticing the inaccuracy in the associated text. The words “for 10 out of the 11” was used twice in the manuscript text, with this instance being the second time and also the source of confusion. Even though in both situations there are 11 cases, these cases are entirely different. The manuscript was therefore revised from “for 10 out of the 11 evaluated cases” to “for 5 out of the 11 evaluated cases”.

We objectively agree that only 5 of the 11 cases have presented improvements of predictive accuracy. We would also like to mention the reduced feasibility of improving the predictive accuracy to below 20%, which is the situation for several cases.

For more discussions regarding the potential of overfitting in ecModels, we would like to suggest the section '3.2 Simulation caveats' in the Supporting Information of the previously published article on GECKO 1.0¹.

Minor comments

What do authors mean by "wild-cards"? (Line 189)

R: We thank the reviewer for noticing this unclear use of the term. First introduced in line 157, the terms "wild card" and "wild-card" were revised to "wildcard". Moreover, the newly added line in response to note 2 clarifies that wildcards match any E.C. subclass.

Reviewer #2 (Expertise: Metabolic modelling):

Domenzain et al., have presented the updated version of GECKO toolbox in this paper and have demonstrated creation of high-quality enzyme constrained models (ecModels) for functional GEMs. The work is of relevance to the constraint-based modeling field. The paper is well-written. The following are some comments related to the work presented in this paper:

R: We thank the reviewer for the feedback and the kind words.

1. The authors mention that additional condition-dependent exchange fluxes of byproducts can be used as constraints in order to limit the feasible solution space. How does the user decide which condition-specific exchange fluxes need to be used in a particular condition? Can the authors support this with an example?

R: We thank the reviewer for the question. Other published work² has demonstrated how a commercially valuable byproduct (*p*-coumaric acid) can serve as an exchange rate constraint, and in their associated Supplementary Table 3 what other physiological parameters may be further included as constraints.

2. Can the authors define what are "active fluxes" in the context of metabolic fluxes?

R: We thank the reviewer for spotting the unclear shorthand "active fluxes". All such occurrences in the manuscript have been replaced with "active reaction fluxes", or "fluxes of active reactions", as appropriate.

3. How does the method handle reactions that are not associated with enzymatic activities? For example, some exchange and transport reactions that do not have any GPRs associated.

R: We thank the reviewer for highlighting the lack of clarity with regards to how reactions that are not associated with enzymatic activities are processed by GECKO 2.0. In accordance with the formalism previously published, ecModels are defined in an irreversible format. Therefore, all reversible reactions are converted to the irreversible format. When no enzymes are associated to a reaction, said reaction will not be further constrained by GECKO, except for flux constraints imposed manually by the user. A comprehensive description

¹ Sánchez, Benjamín J., et al. "Improving the phenotype predictions of a yeast genome-scale metabolic model by incorporating enzymatic constraints." *Molecular systems biology* 13.8 (2017): 935. <https://doi.org/10.15252/msb.20167411>

² Liu, Q., Yu, T., Li, X. et al. "Rewiring carbon metabolism in yeast for high level production of aromatic chemicals." *Nat Commun* 10, 4976 (2019). <https://doi.org/10.1038/s41467-019-12961-5>

of these aspects has been included in the Supporting Information of the previously published article on GECKO 1.0³, section 1.3 'Including enzymes in reactions' and 1.4 'Example with a toy model'.

4. Based on the Kcat distribution in BRENDA, is it possible to predict the minimum cut-off needed for constraining the GEM of other organisms like Mtb, Mus musculus, Arabidopsis etc.?

R: We thank the reviewer for the question regarding the applicability of GECKO 2.0 outside what was presented in the current work. As shown in Suppl. 1, the 3 organisms in question are part of the top 10 organisms by number of reported k_{cat} values (Table S1.4), which indicates there would be sufficient data for our method to work. In addition, we would like to mention that the ecModels container already contains early work towards obtaining an enzyme-constrained model for *Mus musculus*, available transparently at <https://github.com/SysBioChalmers/ecModels/pull/84>. To indicate that such future work is possible, a new sentence was added (after line 470 in the previously submitted manuscript): "Moreover, the implemented automation facilitates the application of the GECKO method to other organisms for which sufficient data is available."

5. In case of multi-omics data available for organisms like yeast and humans, is it possible to integrate that information in the model along with the proteins to constrain the model and obtain biologically meaningful predictions?

R: We thank the reviewer for the insight in using enzyme-constrained models obtained by the GECKO method as a framework for multi-omics data integration. Our previous work⁴ indicates this is possible, as does independent work⁵.

6. How much is the computing time needed for analyzing the ecModels considering the fact that they are of different sizes (reactions, metabolites and proteins)?

R: We thank the reviewer for bringing up the downstream implications of the increased size of the ecModels. It is known that the source GEMs vary in size; consequently, the ecModels vary in size even more, depending on the GPRs. Without constraining the question to a specific ecModel, dataset, toolbox, solver and analysis, it is hard to provide robust time estimates. That being said, running the flux variability analysis shown in Figure 5 locally on a laptop for both growth rates took approximately 13 minutes for *K. marxianus*, 10 minutes for *Y. lipolytica* and 23 minutes for *E. coli*.

Minor comments:

1. The term "eukaryal" is not commonly used term. It is more appropriate to use the term "eukaryote" for *S. cerevisiae*.

R: We thank the reviewer for the language suggestion. Line 152 has been revised as suggested, from "eukaryal organisms" to "eukaryote organisms".

³ Sánchez, Benjamín J., et al. "Improving the phenotype predictions of a yeast genome-scale metabolic model by incorporating enzymatic constraints." *Molecular systems biology* 13.8 (2017): 935. <https://doi.org/10.15252/msb.20167411>

⁴ Sulheim, Snorre, et al. "Enzyme-constrained models and omics analysis of *Streptomyces coelicolor* reveal metabolic changes that enhance heterologous production." *Iscience* 23.9 (2020): 101525. <https://doi.org/10.1016/j.isci.2020.101525>

⁵ Zhou, J., Zhuang, Y. & Xia, J. "Integration of enzyme constraints in a genome-scale metabolic model of *Aspergillus niger* improves phenotype predictions." *Microb Cell Fact* 20, 125 (2021). <https://doi.org/10.1186/s12934-021-01614-2>

2. In line 363-364 the authors mentioned "This suggests that upper bounds on enzyme usages induce differentiated utilization of isoenzymes, reflecting well why isoenzymes have been maintained throughout evolution". Can the authors elaborate more on some examples of isozymes to support their point?

R: We thank the reviewer for the interest in exploring Suppl. file 3 containing results for *K. marxianus*, *Y. lipolytica* and *E. coli*. We are taking the opportunity to explore a random example for *S. cerevisiae* extracted from this supplementary file, showing how isozymes supporting our point can be found in our results.

In Suppl. file 3, in the 'Sce_saturated_enzymes' sheet on row 2 we notice the saturation of enzyme Q07500 / gene YDL085W / enzyme name NDE2. We can then proceed to look in the sheet 'Sce_flux_dist' for the gene 'YDL085W', finding it on row 621 and noticing that it is part of reaction 'r_0770' named 'NADH dehydrogenase, cytosolic/mitochondrial' with the associated gene-reaction rule 'YDL085W or YMR145C'. Based on this gene-reaction rule, we understand that YDL085W and YMR145C are isoenzymes. Next, we can observe the difference in enzyme usage for these enzymes in the ecModel in column names with '_ecM' versus the ecModel with protein constraints in columns names with '_ecP'. Looking at the 'HiT' columns, for the high temperature experimental condition, we notice how the saturated YDL085W facilitated the use of the isozyme YMR145C in the ecP model, which was not the case in the ecM model. This constitutes an example of differentiated utilization of isoenzymes facilitated by the incorporation of proteomics data.

3. Did the authors use fastFVA for the analysis shown in this paper?

R: We thank the reviewer for highlighting the unclarity regarding the FVA method used. Lines 402-406 mention that FVA cannot be performed with COBRA nor RAVEN toolboxes. Consequently, fastFVA, as part of the COBRA toolbox and included in their documentation <https://opencobra.github.io/cobratoolbox/stable/modules/analysis/FVA/fastFVA/index.html>, cannot be used in this work.

Reviewers' Comments:

Reviewer #1:

Remarks to the Author:

Authors have clarified some of my previous questions. However, I think some of them are still unaddressed.

Previous Question 2: How did authors validate the use of a phylogenetic closest distance to infer kcats? Obviously, kcats in GECKO 2.0 are going to be different to the ones in GECKO 1.0. But this is not answering my question.

One strategy might be to separate a number of available kcat entries in Brenda (test data) and apply the hierarchical matching algorithm without them. Finally, compare predicted kcats with available kcats in BRENDA. This could be done for both GECKO 1.0 and GECKO 2.0 and compare the results. I would expect positive results.

Figure 1G provides one single case where GECKO 2.0 is superior to GECKO 1.0. Author provides relative test error for Figure 1G, while in Figure 2 sum of square errors. I recommend being consistent in the performance metrics. In addition, in Figure 2 the results for ECmodel using GECKO 1.0 are not presented.

With respect to my previous Question 5, if proteomics improved 5 out of 11 evaluated cases, is this result statistically significant? I obtain a p-value of 0.12 when applying the binomial test (see below R code), which is not significant.

$1 - \text{pbinom}(5, 11, 1/3) = 0.122085$.

Reviewer #2:

Remarks to the Author:

The authors have presented an upgraded version of the GECKO toolbox and have demonstrated its usefulness in the metabolic modeling field. With more omics data becoming available for various organisms, this toolbox will be useful for generating enzyme constrained models for more organisms. The codes are made available for reproducibility and the methods sound reasonable.

Revision cover letter

with detailed point-by-point responses to the reviewers' comments to the resubmission

Dear Editor,

Thank you very much for the e-mail dated on January 25, 2022, which included the comments of the two reviewers on our manuscript NCOMMS-21-10902. Please find the enclosed revised version of the manuscript "Reconstruction of a catalogue of genome-scale metabolic models with enzymatic constraints using GECKO 2.0". We are grateful to the two experts' follow-up comments, and we propose further corrections for improving the paper. All the raised issues have been addressed, and the re-submitted files have been modified accordingly.

The point-by-point responses we made to the valuable suggestions given by the reviewers are below, where *R*: refers to our response. The original portions of text have been coloured in black to facilitate tracking changes.

Reviewer #1

Authors have clarified some of my previous questions. However, I think some of them are still unaddressed.

R: We thank the reviewer for the attention to detail and we appreciate the opportunity to further clarify our work.

Previous Question 2: How did authors validate the use of a phylogenetic closest distance to infer k_{cat} s? Obviously, k_{cat} s in GECKO 2.0 are going to be different to the ones in GECKO 1.0. But this is not answering my question.

R: We thank the reviewer for the follow-up question on k_{cat} matching. In our previous revision we had stated that " k_{cat} values are merely sorted by, and not inferred from, the phylogenetic distance" and had updated the manuscript accordingly.

In other words, in GECKO 2.0, like in the previous GECKO 1.0, there is no inference of k_{cat} values. Consequently, given the same version of the BRENDA database, the only difference introduced by the new matching algorithm is which of the existing k_{cat} value to choose.

To address this still unclear part of the manuscript, we have changed "where GECKO 1.0 would have chosen available k_{cat} values for any organism, GECKO 2.0 chooses those available" to "where GECKO 1.0 chooses k_{cat} values available in BRENDA regardless of organism, GECKO 2.0 chooses the values available in BRENDA for the phylogenetically closest organism".

We hope that our improved clarifications convey that there is no inference of k_{cat} values in this work. Regardless, k_{cat} inference of is a very interesting subject, and we would like to point the reviewer to relevant work by Heckmann et al. (2018) and Li et al. (2021).

One strategy might be to separate a number of available k_{cat} entries in Brenda (test data) and apply the hierarchical matching algorithm without them. Finally, compare predicted k_{cat} s with available k_{cat} s in BRENDA. This could be done for both GECKO 1.0 and GECKO 2.0 and compare the results. I would expect positive results.

R: We thank the reviewer for the suggestion. However, as clarified above, there is no k_{cat} prediction/inference in our work. Here, we compare the absence of the matching algorithm in GECKO 1.0 against its introduction in GECKO 2.0. The result of this improvement is illustrated in Figure 1E, showing that for the majority of the enzymes, instead of introducing 1 wildcard like in GECKO 1.0, GECKO 2.0 prefers to choose existing k_{cat} values from the same enzyme class.

Figure 1G provides one single case where GECKO 2.0 is superior to GECKO 1.0. Author provides relative test error for Figure 1G, while in Figure 2 sum of square errors. I recommend being consistent in the performance metrics. In addition, in Figure 2 the results for ECmodel using GECKO 1.0 are not presented.

R: We thank the reviewer the comments on consistency. We presume the reviewer is referring to Figure 3A instead of Figure 2. The sum of squared errors was thus removed from Figure 3A, but the values were moved to the figure caption. Moreover, the average relative error was also added to the caption. Additionally, a minor mistake was found and corrected regarding R^2 in the caption of Figure 3A.

One of the main achievements of GECKO 2.0 over GECKO 1.0 is being able to apply the formalism to other organisms besides *S. cerevisiae*. Since GECKO 1.0 has not aimed to, and did not, produce an ecModel for *E. coli*, our manuscript includes only a comparison between the GECKO 2.0-obtained *eciML1515* and the previous *ME-iJL12678* developed by other authors.

With respect to my previous Question 5, if proteomics improved 5 out of 11 evaluated cases, is this result statistically significant? I obtain a p-value of 0.12 when applying the binomial test (see below R code), which is not significant. $1 - \text{pbinom}(5,11,1/3) = 0.122085$.

R: We thank the reviewer for inquiring about the statistical significance of the result and agree with their assessment. While we have not claimed the result to be statistically significant, we understand that the manuscript leaves room for (mis)interpretation. To address this, we have added the following sentence “Although only a trend and not a significant improvement, it would be of interest, in the future, to run further analyses that include more proteomics datasets.”

Reviewer #2

The authors have presented an upgraded version of the GECKO toolbox and have demonstrated its usefulness in the metabolic modeling field. With more omics data becoming available for various organisms, this toolbox will be useful for generating enzyme constrained models for more organisms. The codes are made available for reproducibility and the methods sound reasonable.

R: We thank the reviewer for the time invested in understanding our work and the accompanying manuscript.

References

- Heckmann, D., Lloyd, C. J., Mih, N., Ha, Y., Zielinski, D. C., Haiman, Z. B., Desouki, A. A., Lercher, M. J., & Palsson, B. O. (2018). Machine learning applied to enzyme turnover numbers reveals protein structural correlates and improves metabolic models. *Nat Commun*, 9(1), 5252. <https://doi.org/10.1038/s41467-018-07652-6>
- Li, F., Yuan, L., Lu, H., Li, G., Chen, Y., Engqvist, M. K. M., Kerkhoven, E. J., & Nielsen, J. (2021). Deep learning based kcat prediction enables improved enzyme constrained model reconstruction. <https://doi.org/10.1101/2021.08.06.455417>

Reviewers' Comments:

Reviewer #1:

Remarks to the Author:

Authors have addressed my previous comments. Thank for the clarifications.