# Supplementary material for: Reconstruction of a catalogue of genome-scale metabolic models with enzymatic constraints using GECKO 2.0
Source: Nat Commun. 2022 Jun 30;13:3766. doi: 10.1038/s41467-022-31421-1 (PMC9246944; doi:10.1038/s41467-022-31421-1)
Supplement: Supplementary file 1 — Supplementary information File [file 41467_2022_31421_MOESM1_ESM.pdf]

Supplementary Figures

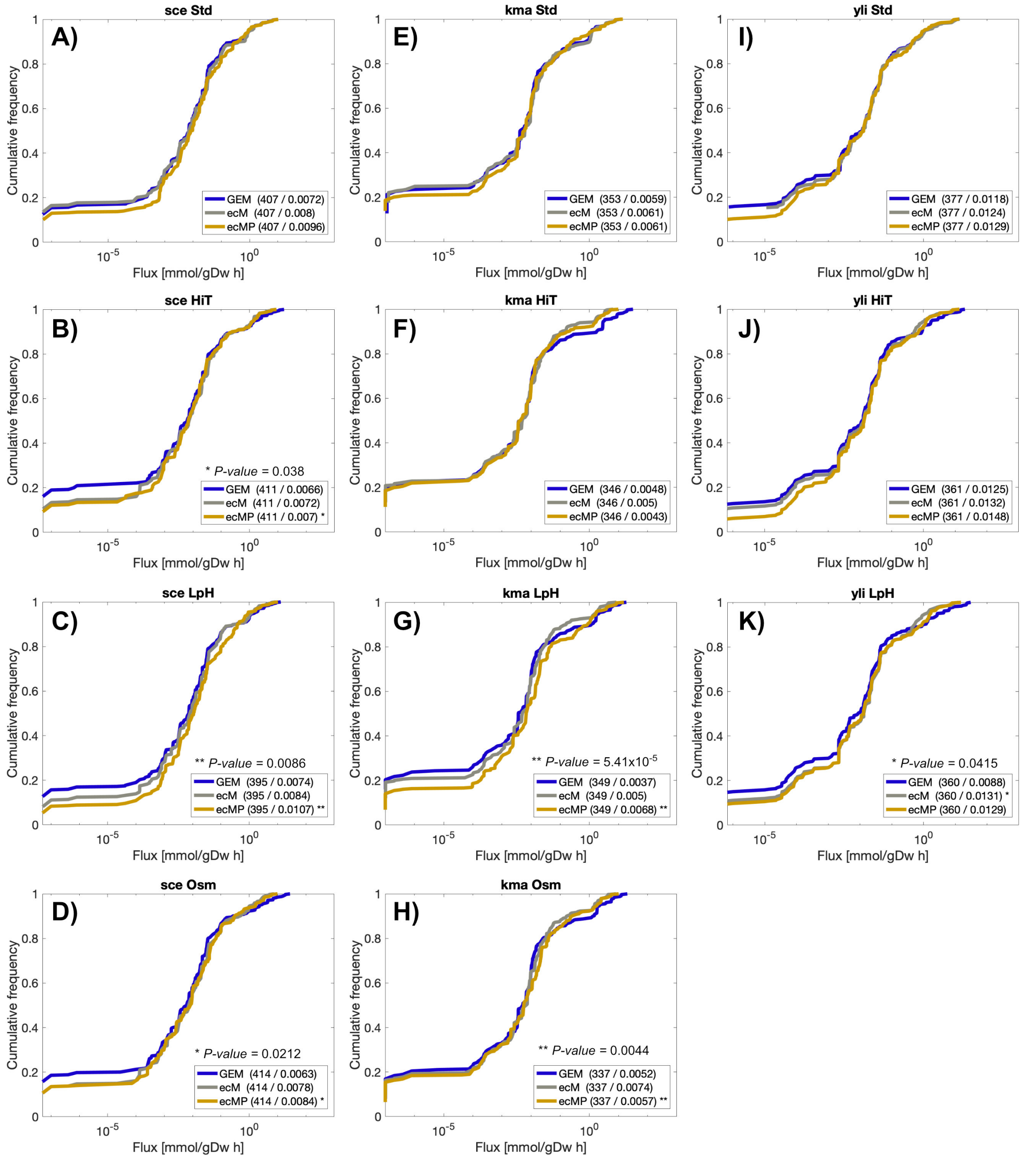

Supplementary Figure 1. Cumulative distributions of metabolic fluxes. Flux distributions of ecModels were mapped to their corresponding reactions in the original GEMs and plotted together as cumulative distributions for all organisms and conditions. **A-D)** Cumulative distributions for *S. cerevisiae* models; **E-H)** Cumulative distributions for *K. marxianus* models; I-K) Cumulative distributions for Y. lipolytica

models. Sample size and median flux values, in mmol/gDwh, are shown within parenthesis for all distributions in all the plots. Statistical significance under a pairwise two-tailed Kolmogorov-Smirnov test between flux distributions for ecModels and their corresponding GEMs are shown as * (0.01 <= *p-value* < 0.05) and ** (*p-value*<0.01). Computed significant *P-values* are indicated for each case. sce – *S. cerevisiae*, kma – *K. marxianus*, yli – *Y. lipolytica*, std – Reference condition, HiT – High temperature condition, LpH – Low pH condition, Osm – Osmotic stress condition, GEM – Genome-scale metabolic model, ecM – ecModel with total protein pool constraint – ecP – ecModel with proteomics constraints. Source data are provided in Data Source File 2.

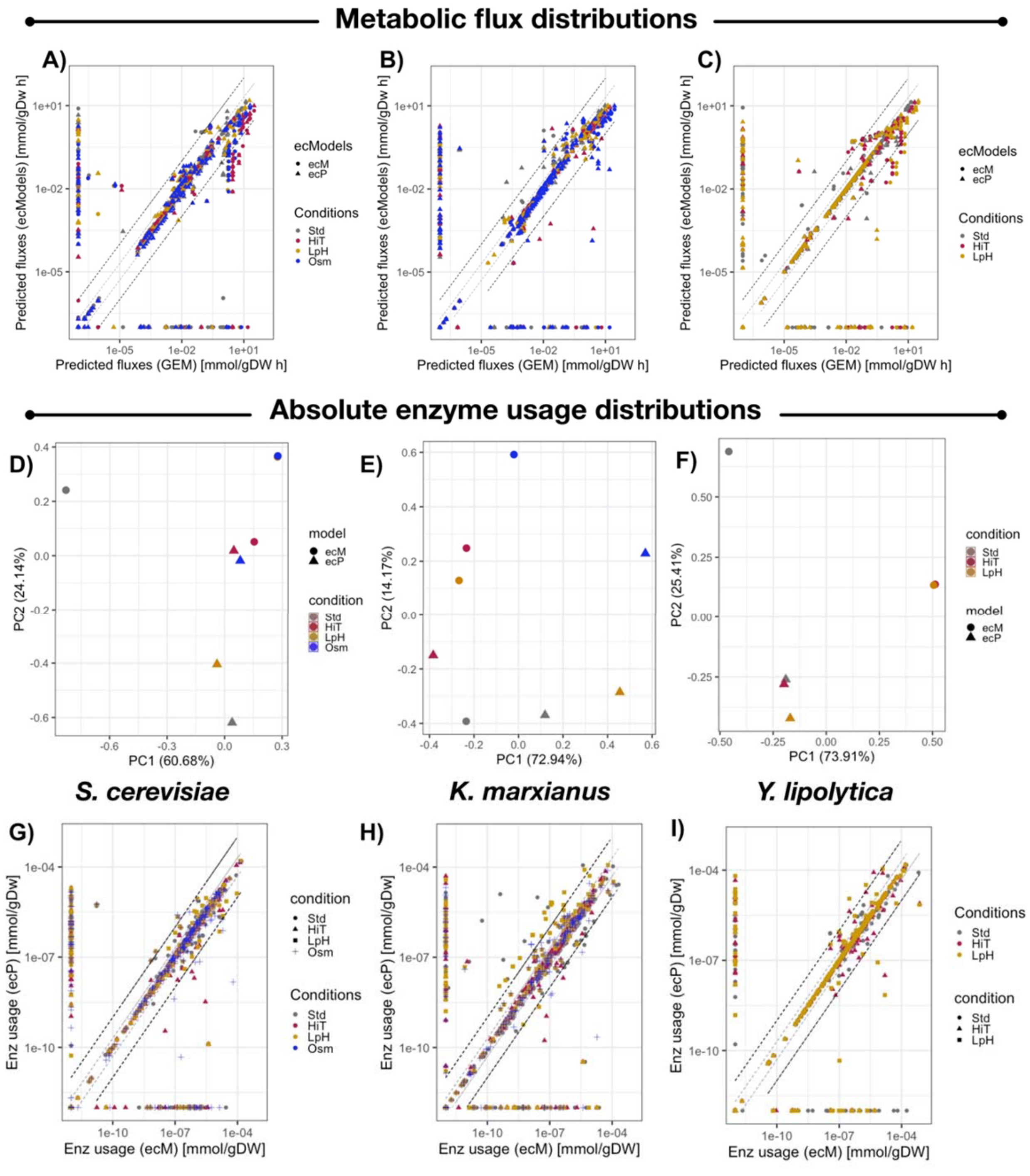

Supplementary Figure 2. Comparison of predicted metabolic fluxes and enzyme usage distributions. Pairwise comparison of metabolic fluxes predicted by GEMs, ecModels and proteomics-constrained ecModels for **A)** *S. cerevisiae*, **B)** *K. marxianus* and **C)** *Y. lipolytica*. Principal component analysis on enzyme usage distributions predicted by ecModels and proteomics-constrained ecModels for **D)** *S.*

cerevisiae, **E)** *K. marxianus* and **F)** *Y. lipolytica* subject to different environmental conditions. Pairwise comparison of enzyme usage profiles in mmol/gDw predicted by ecModels and ecModels with proteomics constraints for **G)** *S. cerevisiae* **H)** *K. marxianus* **I)** *Y. lipolytica*. Grey dashed lines indicate predictions in the interval $0.5 \leq fold\ change \leq 2$, whilst black dashed lines delimit the region of predictions within $0.1 \leq fold\ change \leq 10$, when comparing GEMs to ecModels **(A-C)** and ecModels to ecModels with proteomics data (**G-I**). Std – Reference condition, HiT – High 4niport4ure condition, LpH – Low pH condition, Osm – Osmotic stress condition, GEM – Genome-scale metabolic model, ecM – ecModel with total protein pool constraint – ecP – ecModel with proteomics constraints. Source data are provided in Data Source File 2.

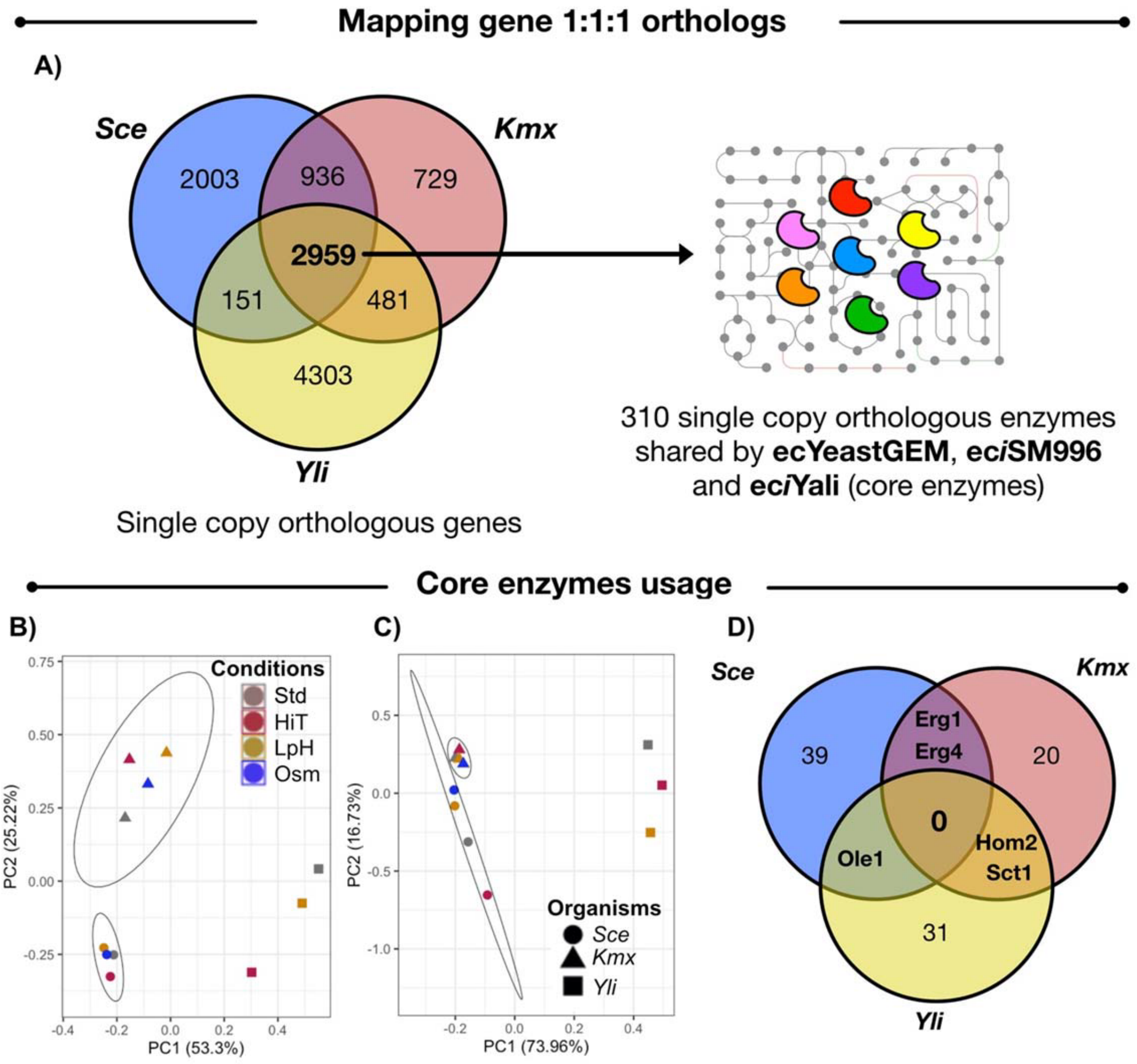

Supplementary Figure 3. Evolutionary conserved enzymes across *S. cerevisiae*, *K. marxianus* and *Y. lipolytica*. **A)** Conservation of protein-coding genes amongst the three budding yeasts. Overlaps indicate number of genes conserved as single copy orthologs between yeast species. The uniprot codes for the 2,959 conserved protein-coding genes amongst the three species were mapped to their corresponding ecModels (ecYeastGEM, ec*i*SM996 and ec*i*Yali), 310 enzymes were found as single copy orthologs across the three ecModels. **B)** Principal component analysis on absolute abundances for the 310 core enzymes across the three yeast species for several experimental conditions. **C)** Principal component analysis on absolute enzyme usages predicted for the 310 core enzymes by ecYeastGEM, ec*i*SM996 and ec*i*Yali for several experimental conditions. **D)** Venn diagram for all core enzymes predicted as highly saturated ($relative\ usage \geq 0.95$) in at least one environmental condition across the three yeast species. Std – Reference condition, HiT – High temperature condition, LpH – Low pH condition, Osm – Osmotic stress condition, Sce – *S. cerevisiae*, Kmx – *K. marxianus*, Yli – *Y. lipolytica*. Source data are provided in Data Source File 2.

Supplementary Methods

Table of Contents

Improved k_{cat} matching algorithm *1*
Estimation of phylogenetic distance between pairs of organisms *2*
Iterative curation of limiting k_{cat} numbers based on parameter sensitivity coefficients *3*
Incorporation of proteomics constraints *3*
Comparative flux variability analysis *5*
Absolute protein quantification *6*
Total protein extraction **6**
Proteins digestion **7**
Mass spectrometry analysis **7**
Estimation of absolute protein abundance values **8**
References *8*

Improved k_{cat} matching algorithm

The k_{cat} matching algorithm in the GECKO toolbox queries kinetic parameters from BRENDA, the largest database available on enzymatic information[1]. However, one of the most important limitations to consider is that such parameters are only available for <10% of the known biochemical reactions[2]. The turnover number assignment to each of the enzymatic reactions present in a GEM is based on a flexible algorithm that allows the incorporation of kinetic parameters even when values for the specific organism and natural substrate of the enzyme are not available. However, as overestimation of microbial growth rates under environmental and genetic perturbations remains one of the main challenges for GEM development, biological relevance of the imposed kinetic constraints plays a crucial role for improving predictive accuracy[3]. In this regard, a global analysis for the reported k_{cat} values on BRENDA (**Supp. file 1**) pointed out the following potential issues.

1. The availability of kinetic parameters is highly heterogeneous, i.e. not all organisms have been studied to the same extent.
2. k_{cat} value distributions showed to be significantly different amongst kingdoms of life, therefore the catalytic activity of enzymes might be phylogenetically constrained.
3. k_{cat} value distributions are highly dependent on the metabolic context. For all kingdoms of life, there are important differences on the distributions for enzymes belonging to different metabolic

pathways groups, being central carbon and energy metabolism enzymes the fastest group (on average) when compared to those involved in amino acid, fatty acid and nucleotide metabolism and secondary and intermediate metabolism.

In order to address the aforementioned limitations, the GECKO k_{cat} matching algorithm was modified aiming to provide a more accurate parameterization of models. A comparison between the introduced and previous hierarchical algorithms is shown in **Table x.**

Table S2.1.- k_{cat} matching algorithms comparison.

**Original k_{cat} matching criteria**	**New criteria**
As a first option, it will try to match the E.C. number, the organism and the corresponding substrate to some k_{cat} annotation in the BRENDA database.	Same as original.
If no match is found, it will try to match the E.C. number and the substrate, but with any organism available.	If no match is found, it will try to match the E.C. number and the substrate, but for the **phylogenetically closest** organism with available values.
If no match is found, it will try to match the E.C. number and the organism, but with any substrate available.	Same as original.
If still no match is found, it will try to match the E.C. number for any organism, and any substrate available.	If no match is found, it will try to match the E.C. number and the organism but looking in **specific activity** values instead of k_{cat} (S.A.*M_{weight} = k_{cat}).
If still no match is found, then it will introduce one wildcard to the E.C. number and attempt all previous 4 steps again.	If still no match is found, it will try to match a k_{cat} value for the E.C. number, any substrate but for the **phylogenetically closest** organism with available values.
	If still no match is found, it will try to match a **specific activity** value for the E.C. number, any substrate but for the **phylogenetically closest** organism available.
	Finally, if still no match is found, then it will introduce one wildcard (WC) to the E.C. number and attempt all previous 6 steps again.

Estimation of phylogenetic distance between pairs of organisms

The phylogenetic distance between organisms is measured as the number of nodes of separation between two organisms in the KEGG taxonomical tree (incorporated as a MATLAB workspace file into the toolbox), this new feature follows from the assumption that kinetic parameters on enzymes have been finely tuned by evolution and are phylogenetically related[4]. The incorporation of specific activity values

increases the parameter coverage and avoids the assignment of a high number of wild cards, making the assignments as close as possible to the original metabolic function of the specific enzyme class.

Iterative curation of limiting k_{cat} numbers based on parameter sensitivity coefficients

Once kinetic parameters and protein pool bounds have been incorporated into the ecModel it is very likely that overconstraining arises due to the intrinsic uncertainty of the incorporated k_{cat} values. For such cases, the module ***kcat_sensitivity_analysis*** flexibilizes the coefficients with a higher control on the simulated objective function value based on parameter sensitivity coefficients given by

$$C_{ij} = \frac{k_{\text{cat}}^{ij}}{v_{\text{obj}}} \frac{\Delta v_{\text{obj}}}{\Delta k_{\text{cat}}^{ij}}$$

in which k_{cat}^{ij} represents the k_{cat} parameter of the enzyme i in reaction j; v_{obj} is the original value in the objective function; Δk_{cat}^{ij} is an induced perturbation in the k_{cat} equivalent to 10-fold increase of its initial value; Δv_{obj} is the change in the objective function after perturbing k_{cat}^{ij}. The magnitude of the induced perturbations was chosen based on the fact that k_{cat} values for a given enzyme class may span several orders of magnitude, as shown by main **Figure 1A.**

The ECCs are ranked in a decreasing way and the enzyme with the coefficient is then selected for a 10-fold k_{cat} increase, based on the assumption that k_{cat} parameters may span orders of magnitude across organisms and substrates even for the same enzyme class. This procedure iterates until the ecModel is able to reach to provided experimental growth rate in the ***getModelParameters.m*** function. Information regarding the flexibilized k_{cat} values, their respective proteins and reactions, ECCs, flexibilized and original k_{cat}
s is saved as a text file in the ***GECKO/model*** folder of the toolbox under the name ***kcat_modifications.txt***.

Incorporation of proteomics constraints

The ***integrate_proteomics*** module in GECKO enables the generation of condition dependent models with proteomics constraints for any given dataset of absolute protein abundances [mmol/ gDw] with **m** replicates for **n** conditions. The data incorporation algorithm consists of the following steps.

1.- For each experimental condition, the abundance values are filtered, excluding proteins that are not present in at least 2/3 of the total number of condition replicates and also noisy measurements

(proteins with relative standard deviation higher than 1 across replicates). Median abundance values ($[E_i]$) and their standard deviations across replicates (δ) are calculated for each protein.

2.- Upper bounds are imposed on the corresponding enzyme usage pseudo-reactions as follows:

$$ub_{e_i} = [E_i] + 1.96 * \delta$$

The addition of $1.96 * \sigma$ accounts for a confidence interval of 0.95 in the protein abundance measurement.

3.- The experimental value for cellular growth rate at which the proteomics samples were obtained is then fixed as lower bound for the biomass pseudo-reaction and measured fluxes on glucose uptake rate and, optionally, byproducts secretion rates are set as upper bounds for their respective exchange reactions (adding a numerical tolerance of 5%).

4.- The remaining total protein pool is constrained by

$$ub_{P_{pool}} = \left(P_{total} - \sum_i M_{w_i} * [E_i]\right) * \sigma * f$$

where P_{total} is the measured total protein content in the cell in g_{prot}/gDw; $M_{w_i}$is the molecular weight of the measured protein *i*; σ represents an average saturation factor for the unmeasured enzymes, assumed as 0.5[5,6]; f accounts for the fraction that the unmeasured protein sector represents out of the total proteome in the cell, this value is calculated by using a paxDB proteome abundance file for the organism of interest as a reference, if no paxDB file is provided then a value of 0.5 is assumed.

5.- Protein abundances are corrected for the oxidative phosphorylation complexes, trying to avoid overconstraining of potentially erroneously measured subunits that might limit the whole pathway. This correction is limited just to this pathway as it is desirable to modify the original dataset the least possible and abundance changes in OxPhos subunits are key to meet the phenotype energy requirements. Medium constraints are set by allowing free uptake of all compounds available in the culture medium and closing the rest of the uptake reactions. Additionally, all the upper bounds for production reactions (secretion of metabolites) are set to 1000 mmol/gDw h.

6.- Using an ***ecModel_batch*** with the same constraints setup, minimal enzyme requirements for the proteins present in the filtered dataset are retrieved from a parsimonious FBA solution vector.

Enzyme abundances that are lower than the minimum requirements calculated by the FBA solution are corrected in the dataset.

7.- A proteomics constrained ***ecModel_prot*** is obtained by the function ***constrainEnzymes.m***. If the model is overconstrained after imposing all the afore mentioned constraints, then the function ***flexibilizeProteins.m*** flexibilizes the top-limiting abundances (based on shadow prices for the measured proteins, given by: $shadow\ prices = \frac{\partial v_{bio}}{\partial ub_{e_i}}$) until the model is able to grow at the provided experimental growth rate. After this, an optimal enzyme usage profile compatible with the provided constraints is obtained and optimal levels are set as upper bounds for the flexibilized protein usages.

8.- The total flexibilized mass of protein is drawn from the remaining protein pool (upper bound for protein_pool_exchange pseudo-reaction) for consistency with mass conservation. Non-growth associated ATP maintenance is fitted according to condition specific experimental data if available (measurements on exchange fluxes of oxygen and CO_2 from the same samples as the proteomics dataset). In the case of chemostat samples such conditions are set by first fixing the growth rate to the experimental value, minimizing the carbon source uptake, fixing its optimal value and then setting the total unmeasured enzymes usage as a new objective to minimize. Each condition-specific model is saved in ***GECKO/models/prot_constrained***.

Comparative flux variability analysis

The function ***comparativeFVA.m*** in the FVA utitilities module provides a fair comparison of flux variability range distributions between a given GEM and its ecModel pair for glucose limited conditions (low dilution rates) and protein limiting regime (batch growth). The procedure for performing the flux variability analysis in this study is summarized by the following steps.

1.- For the chemostat case, a dilution rate of 0.1 h^{-1} was set as both lower and upper bound for the biomass pseudo-reaction (+/- a tolerance value of 0.01%).

2.- The glucose uptake rate is set as an objective to minimize and its optimal value is then also fixed, using the same tolerance.

3.- Additional culture medium constraints are imposed (upper bound for exchange reactions of mineral minimal medium components were set to 1000 mmol/gDw h).

4.- All applied constraints are also applied to the original GEM.

5.- For every reaction that is able to carry a non-zero flux in the original GEM (assessed by the RAVEN toolbox function ***haveFlux.m***) both minimization and maximization are performed for the original GEM.

6.- For the ecModel, such optimizations are performed on the governing pseudo-reaction representing the same original reaction flux (i.e. arm reactions when isoenzymes are present), this is done for both the forward reaction and its reversible counterpart (if present). In order to avoid the introduction of artificial variability, the forward reaction is blocked when the backwards is optimized, and the same is applied to the opposite direction.

7.- For each reaction a flux variability range is given by

$$FV_i = v_i^{max} - v_i^{min}$$

8.- For the ecModel these ranges are given by

$$FV_i = \left(v_i^{max} - v_i^{min}\right) - \left(v_{i,REV}^{max} - v_{i,REV}^{min}\right)$$

For the protein-limiting case, steps 1 and 2 are substituted by the following. Biomass production is maximized with the ecModel and then the optimal value is used to set a lower bound on the same reaction. In order to compare fairly with the original GEM, the same optimal growth rate is fixed as both lower and upper bounds for the biomass pseudo-reaction. A parsimonious flux distribution in which the total protein usage is minimized in the ecModel subject to all of the previous constraints is then obtained, an optimal glucose uptake rate is taken from this flux distribution and fixed for both ecModel and GEM.

Absolute protein quantification

Total protein extraction

Cells samples for *S. cerevisiae, Y. lipolytica* and *K. marxianus* were washed and suspended in 3 ml of lysis buffer containing: 6 M urea (Sigma, U5378), 2M thiourea (Sigma, T8656), 5mM dithiothreitol

(DTT, Sigma, D0632) and 0.1 M TRIS-HCl pH=8. Cell lysis occurred in a cell disruptor (Constant systems Ltd, One shot model) at 2.4 Kbars and supernatant was recovered by centrifugation (15 min at 4000 g, 4° C). Total protein concentrations were measured according to the 2D Quant kit protocol (GE Healthcare Life Sciences, 80-6483-56).

Proteins digestion

Digestion was performed on excised bands from SDS-PAGE short-migration (1x1 cm lanes, Invitrogen, NP321BOX) gel gradient. Reduction was carried in DTT solution at 10 mM for 30 minutes at 56 °C. The extracts were alkylated with iodoacetamide solution at 55 mM for 45 min in darkness at room temperature (RT). Samples were first digested for 3 h at 37 °C by adding 300 ng of Lysyl-Endopeptidase (Wako, 125-05061). Then, a second digestion was performed with 300 ng of sequencing-grade modified trypsin (Promega) overnight at 37 °C. Supernatants were recovered and the peptides were extracted with a mixture of 0.5% trifluoroacetic acid (TFA) and 50% acetonitrile (ACN) in water. Extracted tryptic peptides were vacuum dried and resuspended in 75 µl of loading buffer containing 0.08% (v/v) of TFA and 2% (v/v) of ACN in water for mass spectrometry (MS) analysis. Simultaneously, the Universal Protein Standard 2 (UPS2, Sigma) was digested in a similar fashion to the experimental samples. The extracted tryptic peptides from UPS2 were vacuum dried and resuspended in 25 µl of loading buffer. We took 1.5-µl samples from the mixture of UPS2 digested peptides (424 ng/µl) and spiked them into 7.5 µl of each of the bulk samples (200 ng yeast peptides/µl) at a ratio of 1:2.35 (UPS2:yeast).

Mass spectrometry analysis

MS analyses were performed on a Dionex U3000 RSLC coupled to an Orbitrap Fusion™ Lumos™ Tribrid™ mass spectrometer (Thermo Fisher Scientific) using a packed column Aclaim™ PepMap™, 75 µm x 500 mm, C18, 3 µm, 100 Å, (Thermo Fisher Scientific). Buffer A consisted of 0.1% formic acid in 2% ACN and buffer B of 0.1 % formic acid in 80% ACN. The peptide separation analysis was achieved at 300 nL/min with a linear gradient from 1 to 35% buffer B for 160 min and 35% to 50% for 10 min. One run took 195 min including the regeneration step at 98 % buffer B. Ionization (1.6 kV ionization potential) and capillary transfer (275°C) were performed with a liquid junction and a capillary probe (SilicaTip™ Emitter, 10 µm, New Objective). MS/MS analysis was performed in Data Dependent Acquisition mode, with a top speed cycle of 3 s for the most intense double or multiple charged precursor ions. Ions in each MS scan over threshold 50,000 were selected for fragmentation (MS2). The mass spectrometer acquisition settings were set as follows. Full MS scan in Orbitrap (scan range [m/z] = 400–

1600) with a resolution of 120,000 (AGC target = 5 x 105, max. injection time of 100 ms, data type = centroid). The dynamic exclusion within 10 ppm during 60 s and the intensity threshold was fixed at 5 x 104. And MS/MS using High Collision Dissociation (HCD) in the Orbitrap with resolution of 15,000 (30% collision energy, AGC target of 5.0×10^4 and max. injection time = 54 ms). Polysilaxolane ions m/z 445.12002, 519.13882 and 593.15761 were used for internal calibration.

Estimation of absolute protein abundance values

Normalized Spectral Abundance Factor (NSAF)[7] values were calculated for all detected proteins based on MS/MS data for all detected proteins. The NSAF values obtained from UPS2 proteins in bulk samples were used to determine the suitable regression curves that allowed the conversion from relative protein abundance into absolute terms. The obtained linear regression parameters are shown in **Table S2.2**. This enabled estimation of protein concentrations (Q_i) in the analyzed bulk samples, which consisted of concentrated mixes of peptides, in units of mmol/$g_{protein}$. In order to obtain specific protein abundances, referred to the original cells dry weight, total protein content in the initial biological samples was measured by using the Lowry method for protein quantification[8]. Finally, absolute protein abundance values were multiplied by the total protein content in their respective samples, yielding values in units of mmol/gDw for consistency with the ecModels units for protein usages.

Table S2.2.- Calibration parameters for protein abundance estimation.

Strain	Equation	r^2	Detected UPS2 proteins
S. cerevisiae CEN.PK113	$\log10(Q_i) = 0.666 * \log10(NSAF_i) – 3.48$	0.901	25
K. marxianus CBS6556	$\log10(Q_i) = 0.619 * \log10(NSAF_i) – 3.33$	0.870	27
Y. lipolytica W29	$\log10(Q_i) = 0.610 * \log10(NSAF_i) – 3.51$	0.874	27

References

1. Jeske, L., Placzek, S., Schomburg, I., Chang, A. & Schomburg, D. BRENDA in 2019: A European ELIXIR core data resource. *Nucleic Acids Res.* (2019). doi:10.1093/nar/gky1048
2. Davidi, D. & Milo, R. Lessons on enzyme kinetics from quantitative proteomics. *Current Opinion in Biotechnology* **46**, 81–89 (2017).
3. Price, N. D., Reed, J. L. & Palsson, B. Genome-scale models of microbial cells: Evaluating the

consequences of constraints. *Nat. Rev. Microbiol.* **2**, 886–897 (2004).

4. Bar-Even, A. *et al.* The moderately efficient enzyme: Evolutionary and physicochemical trends shaping enzyme parameters. *Biochemistry* **50**, 4402–4410 (2011).
5. Nilsson, A. & Nielsen, J. Metabolic Trade-offs in Yeast are Caused by F1F0-ATP synthase. *Sci. Rep.* **6**, 1–11 (2016).
6. Sánchez, B. J. *et al.* Improving the phenotype predictions of a yeast genome□scale metabolic model by incorporating enzymatic constraints. *Mol. Syst. Biol.* **13**, 935 (2017).
7. Zybailov, B. *et al.* Statistical analysis of membrane proteome expression changes in Saccharomyces cerevisiae. *J. Proteome Res.* (2006). doi:10.1021/pr060161n
8. Waterborg, J. H. & Matthews, H. R. The Lowry method for protein quantitation. *Methods in molecular biology (Clifton, N.J.)* **32**, (1994).

BRENDA kinetic data analysis

Ivan Domenzain

June 2022

Contents

Summary **1**

1. Loading data, scripts and packages **1**

1.1 Loading packages . . . 1

1.2 Loading R scripts for this study . . . 1

1.3 Loading enzyme data from BRENDA database . . . 1

2. Preprocess data **2**

2.1 Extend dataset with specific activity values . . . 2

2.2 Get enzymes information (pathways, genes) from KEGG ftp . . . 2

3. Data analysis **5**

Data composition . . . 5

3.1.1 Data composition per enzyme classes . . . 5

3.1.2 Data composition per organism . . . 7

3.1.3 Data composition per metabolic groups . . . 9

3.2 Data dispersion . . . 11

3.3 k_{cat} distributions . . . 14

3.3.1 k_{cat} distributions per enzymes family . . . 14

3.3.2 k_{cat} distributions per metabolic context . . . 17

3.3.3 k_{cat} distributions per organism Kingdoms . . . 18

3.3.4 k_{cat} distributions per metabolic pathways groups per organism Kingdoms . . . 21

Summary

This is a global statistical study of turnover numbers (k_{cat} parameters) for enzymatic reactions reported in the BRENDA database.

1. Loading data, scripts and packages

1.1 Loading packages

```
options(repos = c(
  yihui = 'https://yihui.r-universe.dev',
  CRAN = 'https://cloud.r-project.org'
))

install.packages('xfun')
library(tidyr)
library(dplyr)
library(plyr)
library(knitr)
library(magick)
library(gridExtra)
library(VennDiagram)
library(kableExtra)
library(ggplot2)
library(ggrepel)
library(viridis)
library(tinytex)
```

1.2 Loading R scripts for this study

```
read_chunk('loadData.R')
read_chunk('preprocessData.R')
read_chunk('analyseData.R')
read_chunk('plotData.R')
```

1.3 Loading enzyme data from BRENDA database

All available k_{cat} numbers, specific activities and molecular weights of enzymes have been retrieved from the BRENDA database, using the provided SOAP methods for python in the BRENDA website. k_{cat} numbers entries correspond to measurements that have been performed for a specific EC number, substrate and organism of origin; the same applies to specific activities, however such parameters are not reported in a substrate specific way; Molecular weights are reported for specific pairs of EC number - organism of origin.

Parameters entries for mutant and recombinant enzymes have been discarded, more details of the retrieval steps are described in the python scripts stored in the directory geckomat/brenda_parser of the GECKO toolbox.

- Loading k_{cat} values [1/s]

- Loading Specific Activity values [umol/min/mg]
- Loading MWeights values from BRENDA [mmol/mg]
- Loading enzyme data from KEGG

2. Preprocess data

2.1 Extend dataset with specific activity values

Get extra k_{cat} values from Specific activities and molecular weights data using the relationship: $k_{cat} = SA * M_{weight}$

The original k_{cat} dataset consisted of:

```
## [1] "30162 entries for 3019 different EC numbers"
```

The extended dataset consists of:

```
## [1] "38280 entries for 4130 different EC numbers"
```

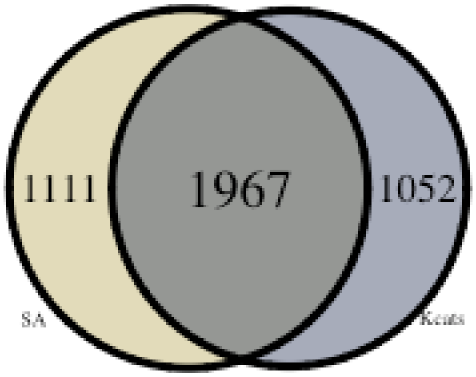

Figure S1.1: Source of unique EC numbers in the dataset

From here onwards, the term dataset will refer to the extended dataset obtained by merging k_{cat} values with those obtained from the specific activities dataset.

2.2 Get enzymes information (pathways, genes) from KEGG ftp

In this study, kinetic parameters distributions are going to be analysed for different enzyme groups, based on enzyme classes, host organism's taxonomy and metabolic context. For the latter, the KEGG pathways classification is used for identifying those enzyme entries in the dataset that have been annotated as part of the following groups:

- Carbohydrates and Energy Metabolism (CEM)

- Amino acids and Lipids Metabolism (ALM)
- Intermediate and Secondary Metabolism (ISM)
- Others

The KEGG database contains pathways and genes information for:

```
## [1] "4921 different EC numbers"
```

A venn diagram for the EC number entries available in BRENDA and KEGG databases is shown below:

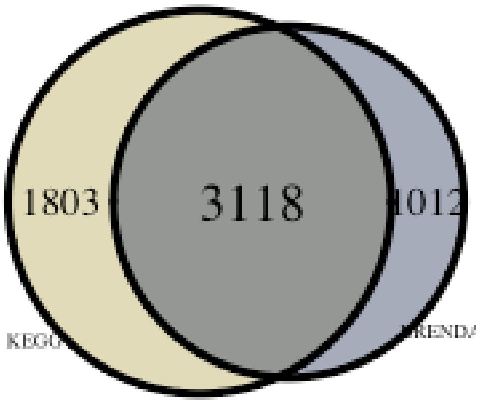

Figure S1.2: Unique EC numbers in BRENDA and KEGG.

The KEGG database provides information that links each EC number entry to all of the pathways in which it might participate. This allows to add a metabolic subgroup to each of the entries in the extended dataset used in this study. Therefore, 3,118 unique EC numbers in the dataset can be classified by metabolic context groups (metGroups).

Note: The EC numbers not present in KEGG are annotated as part of the group 'Others' for this study.

The classified pathways are:

pathways	group
ec00010 Glycolysis / Gluconeogenesis	CEM
ec00020 Citrate cycle (TCA cycle)	CEM
ec00030 Pentose phosphate pathway	CEM
ec00040 Pentose and glucuronate interconversions	CEM
ec00051 Fructose and mannose metabolism	CEM
ec00052 Galactose metabolism	CEM
ec00053 Ascorbate and aldarate metabolism	CEM
ec00500 Starch and sucrose metabolism	CEM
ec00520 Amino sugar and nucleotide sugar metabolism	CEM
ec00620 Pyruvate metabolism	CEM
ec00630 Glyoxylate and dicarboxylate metabolism	CEM
ec00640 Propanoate metabolism	CEM
ec00650 Butanoate metabolism	CEM
ec00660 C5-Branched dibasic acid metabolism	CEM

pathways	group
ec00562 Inositol phosphate metabolism	CEM
ec00190 Oxidative phosphorylation	CEM
ec00195 Photosynthesis	CEM
ec00196 Photosynthesis - antenna proteins	CEM
ec00710 Carbon fixation in photosynthetic organisms	CEM
ec00720 Carbon fixation pathways in prokaryotes	CEM
ec00680 Methane metabolism	CEM
ec00910 Nitrogen metabolism	CEM
ec00920 Sulfur metabolism	CEM
ec00061 Fatty acid biosynthesis	ALM
ec00062 Fatty acid elongation	ALM
ec00071 Fatty acid degradation	ALM
ec00072 Synthesis and degradation of ketone bodies	ALM
ec00073 Cutin, suberine and wax biosynthesis	ALM
ec00100 Steroid biosynthesis	ALM
ec00120 Primary bile acid biosynthesis	ALM
ec00121 Secondary bile acid biosynthesis	ALM
ec00140 Steroid hormone biosynthesis	ALM
ec00561 Glycerolipid metabolism	ALM
ec00564 Glycerophospholipid metabolism	ALM
ec00565 Ether lipid metabolism	ALM
ec00600 Sphingolipid metabolism	ALM
ec00590 Arachidonic acid metabolism	ALM
ec00591 Linoleic acid metabolism	ALM
ec00592 alpha-Linolenic acid metabolism	ALM
ec01040 Biosynthesis of unsaturated fatty acids	ALM
ec00230 Purine metabolism	ALM
ec00240 Pyrimidine metabolism	ALM
ec00250 Alanine, aspartate and glutamate metabolism	ALM
ec00260 Glycine, serine and threonine metabolism	ALM
ec00270 Cysteine and methionine metabolism	ALM
ec00280 Valine, leucine and isoleucine degradation	ALM
ec00290 Valine, leucine and isoleucine biosynthesis	ALM
ec00300 Lysine biosynthesis	ALM
ec00310 Lysine degradation	ALM
ec00220 Arginine biosynthesis	ALM
ec00330 Arginine and proline metabolism	ALM
ec00340 Histidine metabolism	ALM
ec00350 Tyrosine metabolism	ALM
ec00360 Phenylalanine metabolism	ALM
ec00380 Tryptophan metabolism	ALM
ec00400 Phenylalanine, tyrosine and tryptophan biosynthesis	ALM
ec00410 beta-Alanine metabolism	ISM
ec00430 Taurine and hypotaurine metabolism	ISM
ec00440 Phosphonate and phosphinate metabolism	ISM
ec00450 Selenocompound metabolism	ISM
ec00460 Cyanoamino acid metabolism	ISM
ec00471 D-Glutamine and D-glutamate metabolism	ISM
ec00472 D-Arginine and D-ornithine metabolism	ISM
ec00473 D-Alanine metabolism	ISM
ec00480 Glutathione metabolism	ISM
ec00510 N-Glycan biosynthesis	ISM

pathways	group
ec00513 Various types of N-glycan biosynthesis	ISM
ec00512 Mucin type O-glycan biosynthesis	ISM
ec00515 Mannose type O-glycan biosynthesis	ISM
ec00514 Other types of O-glycan biosynthesis	ISM
ec00532 Glycosaminoglycan biosynthesis - chondroitin sulfate / dermatan sulfate	ISM
ec00534 Glycosaminoglycan biosynthesis - heparan sulfate / heparin	ISM
ec00533 Glycosaminoglycan biosynthesis - keratan sulfate	ISM
ec00531 Glycosaminoglycan degradation	ISM
ec00563 Glycosylphosphatidylinositol (GPI)-anchor biosynthesis	ISM
ec00601 Glycosphingolipid biosynthesis - lacto and neolacto series	ISM
ec00603 Glycosphingolipid biosynthesis - globo and isoglobo series	ISM
ec00604 Glycosphingolipid biosynthesis - ganglio series	ISM
ec00540 Lipopolysaccharide biosynthesis	ISM
ec00550 Peptidoglycan biosynthesis	ISM
ec00511 Other glycan degradation	ISM
ec00730 Thiamine metabolism	ISM
ec00740 Riboflavin metabolism	ISM
ec00750 Vitamin B6 metabolism	ISM
ec00760 Nicotinate and nicotinamide metabolism	ISM
ec00770 Pantothenate and CoA biosynthesis	ISM
ec00780 Biotin metabolism	ISM
ec00785 Lipoic acid metabolism	ISM
ec00790 Folate biosynthesis	ISM
ec00670 One carbon pool by folate	ISM
ec00830 Retinol metabolism	ISM
ec00860 Porphyrin and chlorophyll metabolism	ISM
ec00130 Ubiquinone and other terpenoid-quinone biosynthesis	ISM

3. Data analysis

Data composition

3.1.1 Data composition per enzyme classes

Not all of the enzyme classes are of the same interest for the scientific community. Counting the number of data entries available for every unique EC number allows the identification of the top studied enzyme classes.

The top ten represented EC numbers in the dataset are:

Table S1.2: Top10 enzyme classes by number of reported Kcat values

ECnumbers	Entries	% of total entries
EC3.5.2.6	581	1.52 %
EC3.1.1.1	335	0.88 %
EC1.1.1.1	316	0.83 %
EC3.2.1.21	300	0.78 %
EC3.4.21.4	270	0.71 %
EC2.3.2.5	259	0.68 %
EC3.4.21.83	208	0.54 %
EC1.1.99.18	192	0.5 %
EC1.10.3.2	168	0.44 %
EC3.4.21.35	165	0.43 %

Actually, most of the enzyme classes are poorly represented in the dataset, with at least 50% percent of the total number of unique EC numbers having less than 4 reported k_{cat} values (for different organisms and substrates).

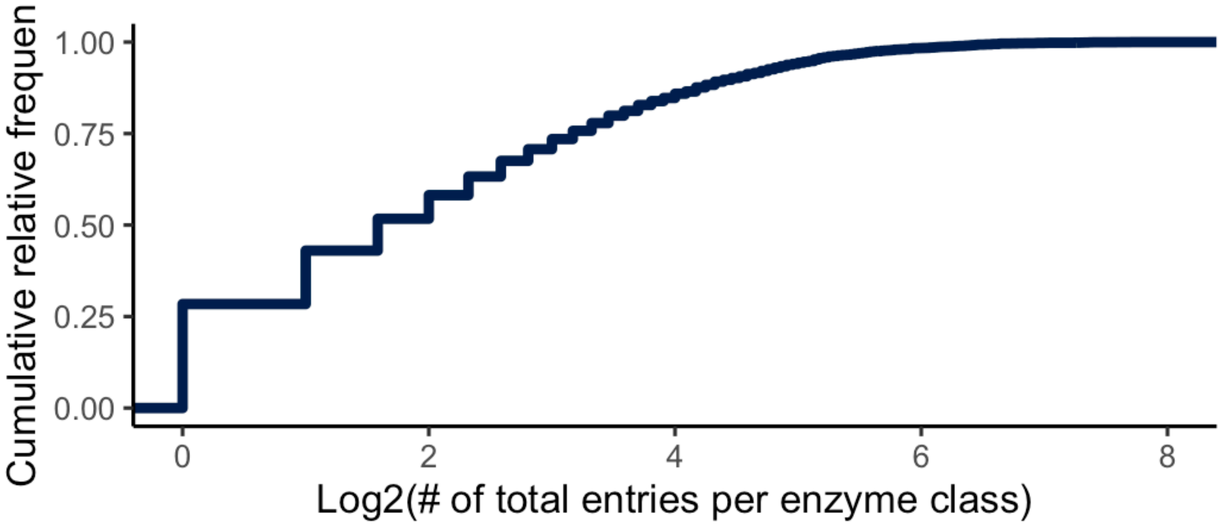

Figure S1.3: Number of entries per enzyme class.

The dataset also presents heterogeneity when it comes to the number of reported parameters for the different families of enzyme classes (top-level EC numbers), being hydrolases (EC3.x.x.x) and oxidoreductases (EC1.x.x.x) the most represented families, comprising almost 70% of the total number of entries.

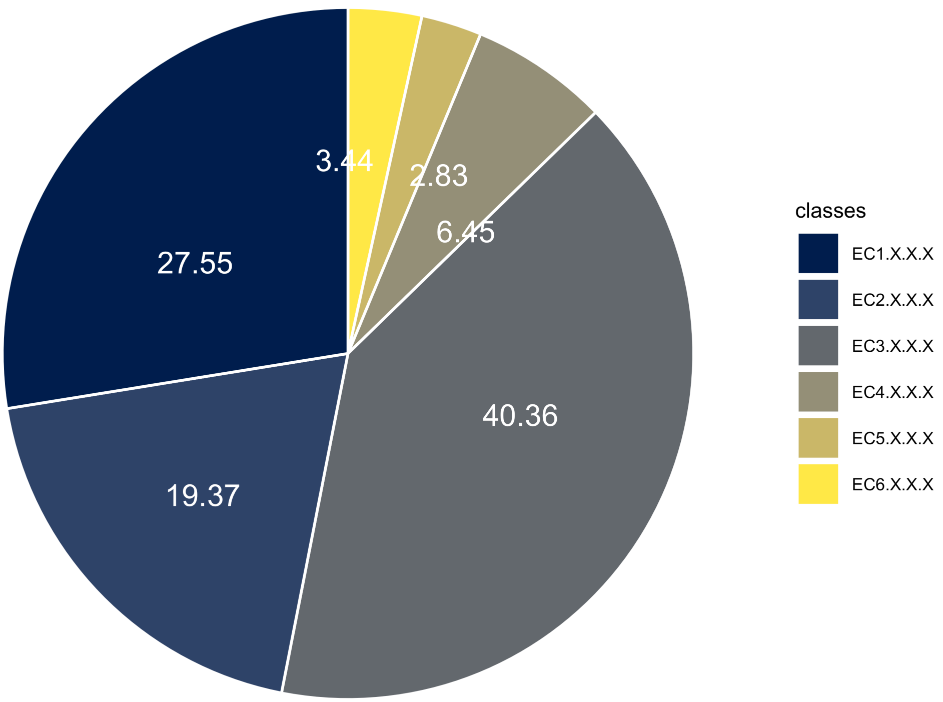

Figure S1.4: Dataset composition by enzyme classes

Table S1.3: Average number of entries per enzyme class for the different enzyme families.

Top-Level_ECnumber	Enzymes_family	Average_entries_per_ECnumber
EC1.X.X.X	Oxidoreductases	8
EC2.X.X.X	Transferases	6
EC3.X.X.X	Hydrolases	14
EC4.X.X.X	Lyases	5
EC5.X.X.X	Isomerases	6
EC6.X.X.X	Ligases	8

3.1.2 Data composition per organism

The different organisms have been grouped into five different **"Kingdoms"** according to the KEGG taxonomy information:

- Eukaryotes//Animals
- Eukaryotes//Plants
- Eukaryotes//Protists
- Eukaryotes//Fungi
- Prokaryotes//Bacteria
- Prokaryotes//Archaea

Number of reported k_{cat} entries per organism:

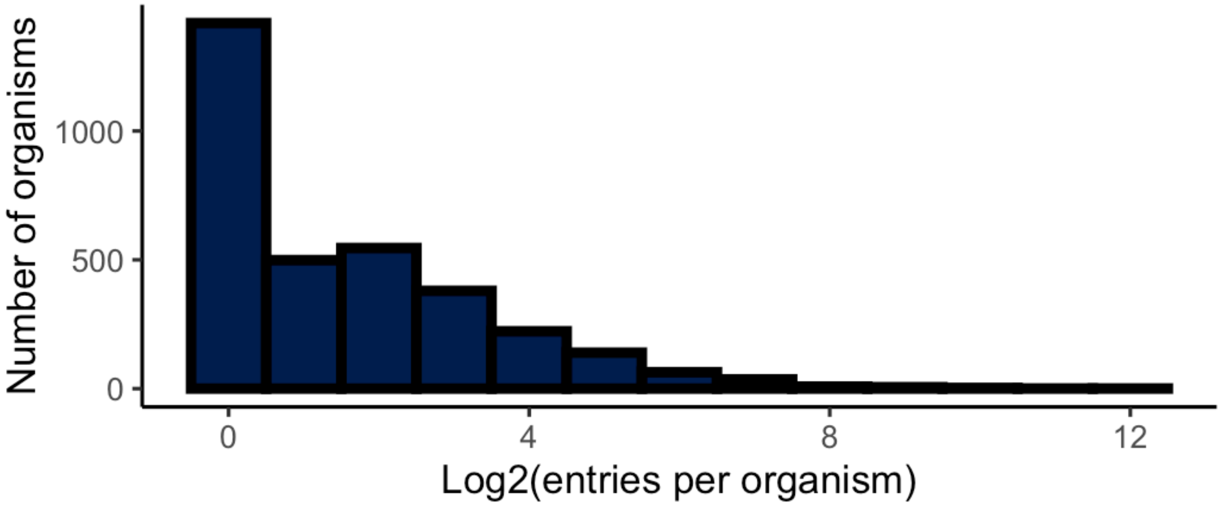

Figure S1.5: Number of entries per organism.

Table S1.4: Top10 organisms by number of reported Kcat values

Organism	Kingdom	Entries	% of total entries
Homo sapiens	Animals	4212	11 %
Escherichia coli	Bacteria	2131	5.57 %
Rattus norvegicus	Animals	1384	3.62 %
Saccharomyces cerevisiae	Fungi	734	1.92 %
Sus scrofa	Animals	733	1.91 %
Bos taurus	Animals	710	1.85 %
Mus musculus	Animals	654	1.71 %
Arabidopsis thaliana	Plants	495	1.29 %
Mycobacterium tuberculosis	Bacteria	475	1.24 %
Pseudomonas putida	Bacteria	377	0.98 %

Representation of the different organism Kingdoms in the analysed dataset

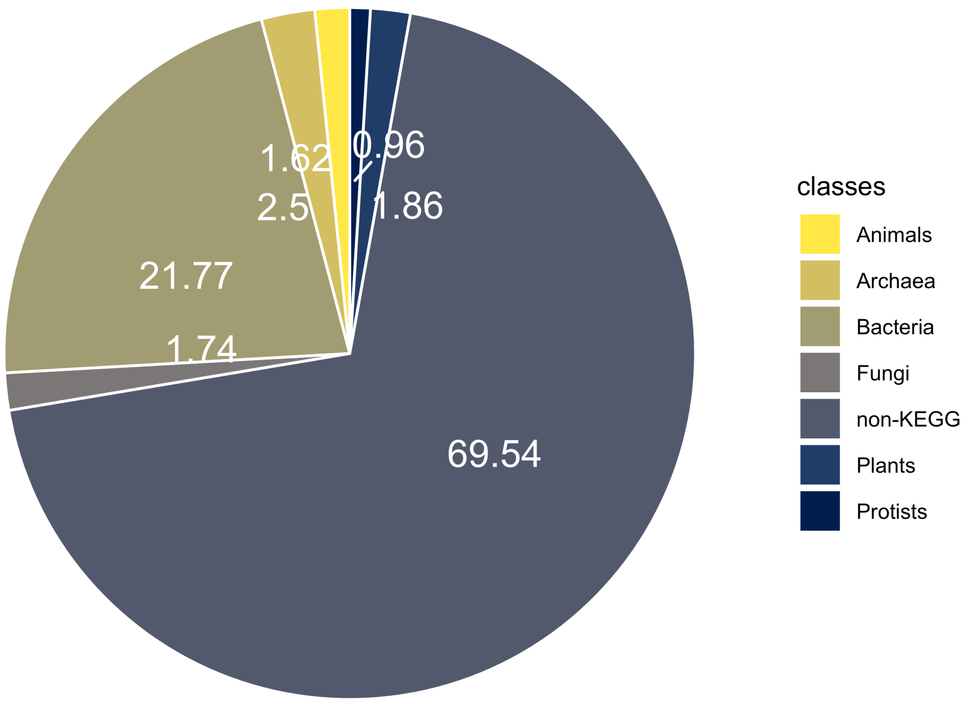

Figure S1.6: Dataset composition by organisms Kingdom

As it has been shown, the studied dataset is biased towards certain well-studied organisms, letting most of the included organisms with a very poor representation in the k_{cat} dataset.

3.1.3 Data composition per metabolic groups

Representation of the different metabolic groups in the analysed dataset.

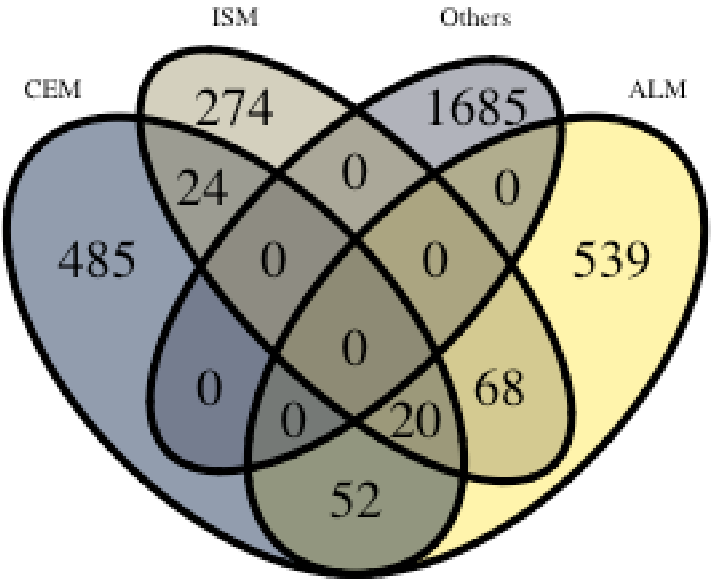

Figure S1.7: Unique EC numbers in the dataset by metabolic subgroup

Unique EC numbers per metabolic groups for the different organism Kingdoms.

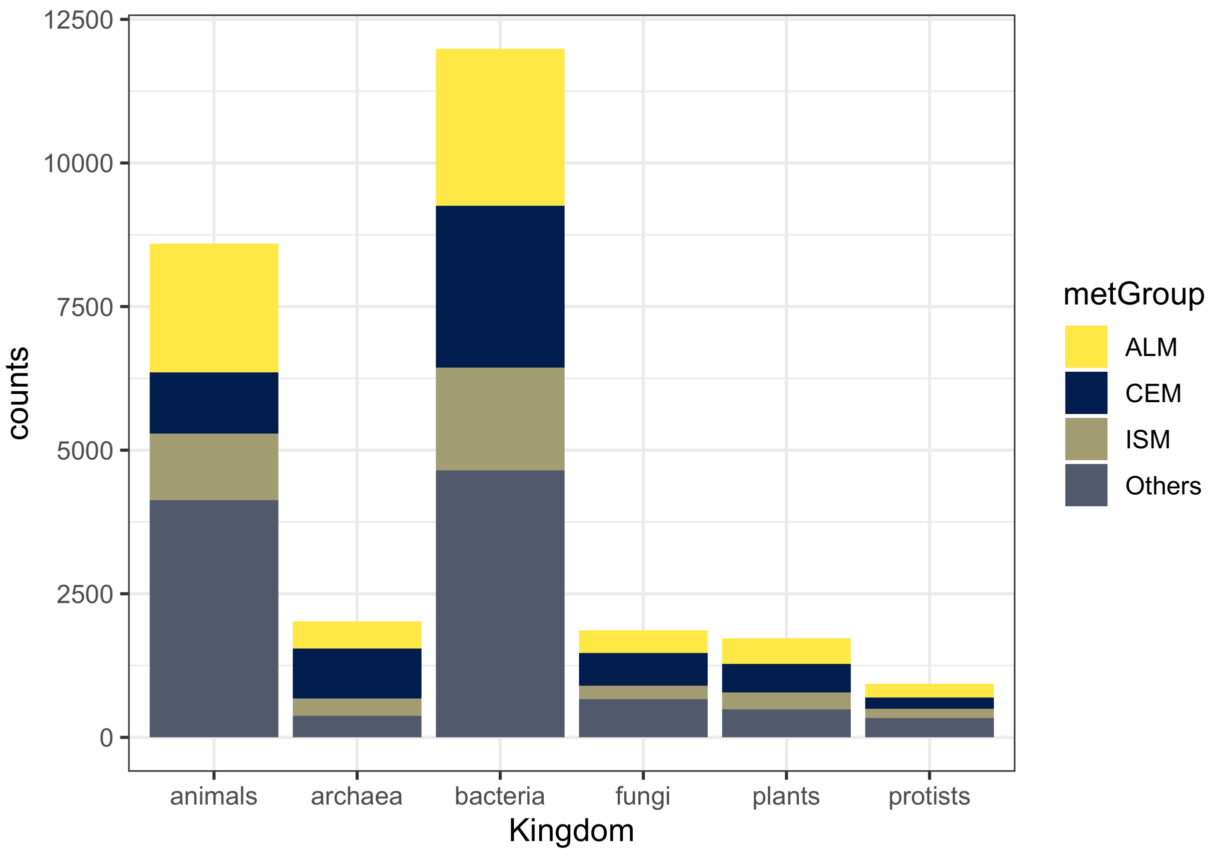

The dataset is not just heterogeneous regarding its composition by organism kingdoms, but also within each of these kingdoms, different metabolic groups of enzymes have been studied in a diverse way, showing a majority of reported values for CEM and ALM enzyme classes for most of the cases.

3.2 Data dispersion

It is said that evolution has shaped kinetic parameters in a very specific way, differentiating catalytic activities by metabolic context, phylogenetic origin and substrate specificity. If such specialization exists for a big dataset as this one, then a wide spanning of reported values can be expected.

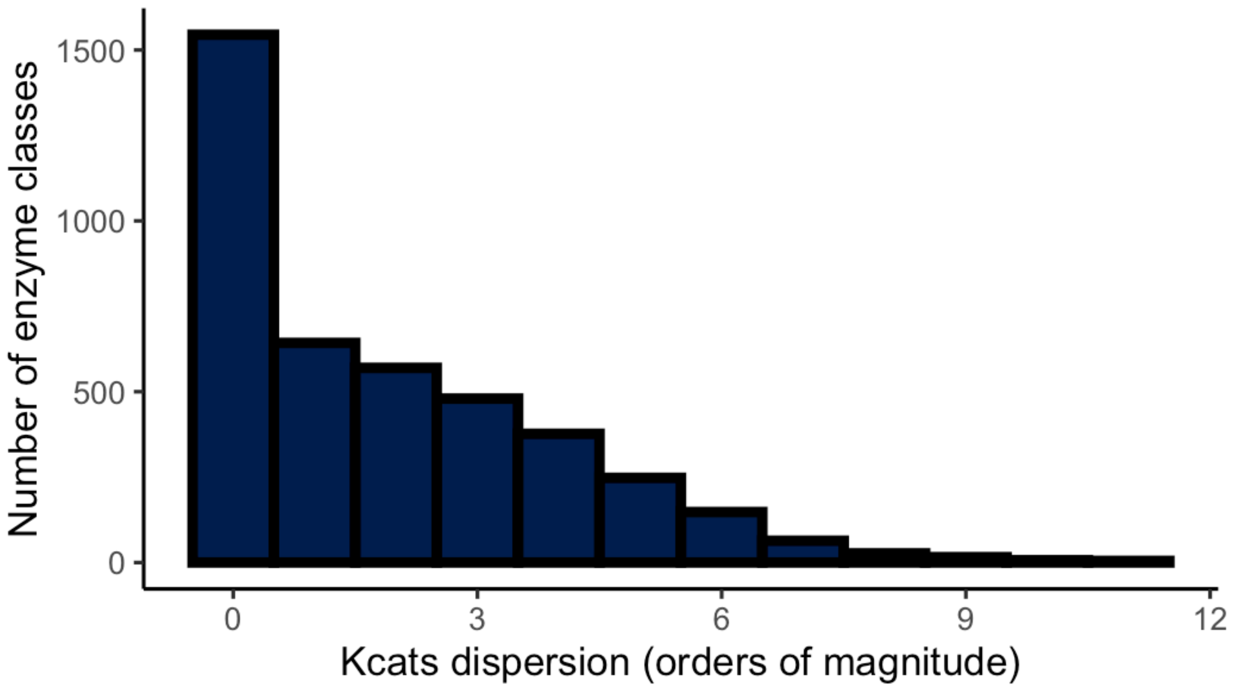

Figure S1.8: Kcats dispersion per EC number.

The previous histogram shows how most of the studied enzyme classes present a very narrow spanning of their reported k_{cat} values, however few enzyme classes (EC numbers) with a surprisingly wide spanning of catalytic activities, (**11 orders of magnitude!**) can also be found in the dataset.

However, it is also important to analyze how large is the variance of a given distribution related to its characteristic values. For this, a normalized dispersion is metric, which here will be called as "spreading" and is defined as:

$spreading = log_{10}(Median)/[log_{10}(Max) - log_{10}(Min)]$.

This metric allows to explore how wide or narrow a distribution is compared to its median value. A value lower than 1 means that the distribution spanning is higher than its median value (in terms of orders of magnitude).

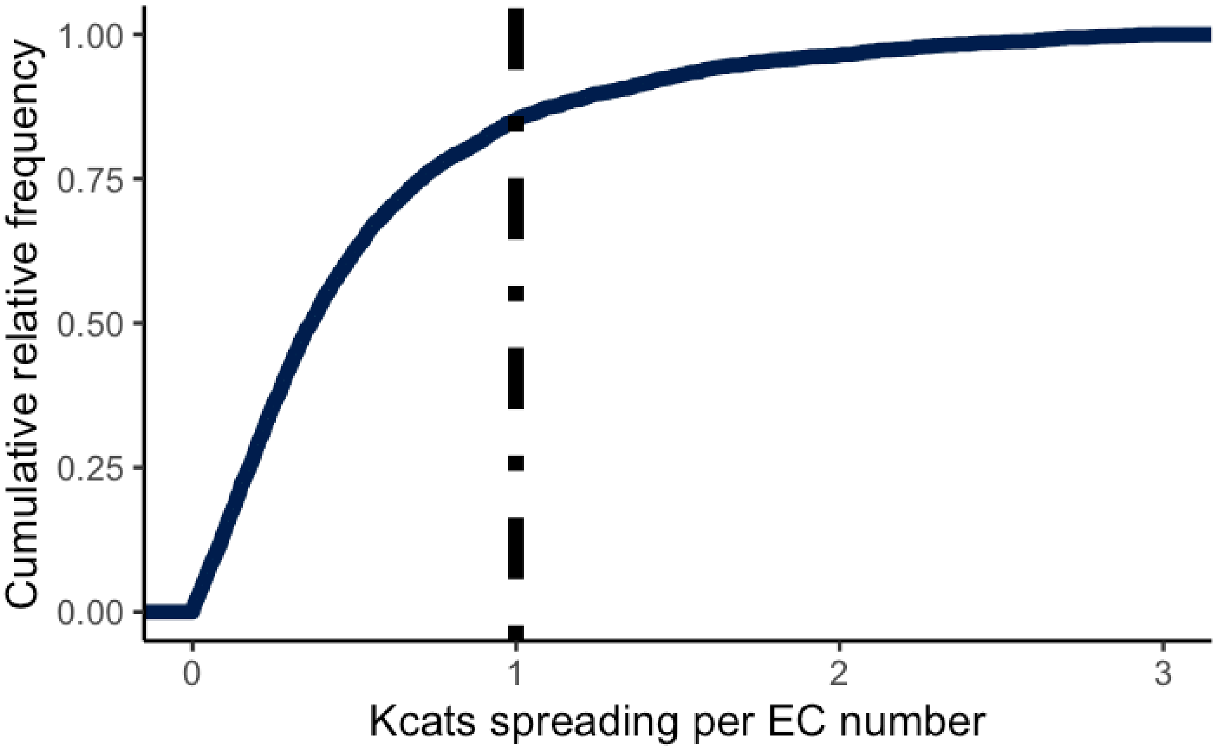

Figure S1.9: Kcats spreading per EC number.

This cumulative distribution shows that around 20% of the enzyme classes show very wide distributions of k_{cat} values in which a median or mean value cannot be considered as representative of the distribution due to its large spanning.

3.3 k_{cat} distributions

3.3.1 k_{cat} distributions per enzymes family

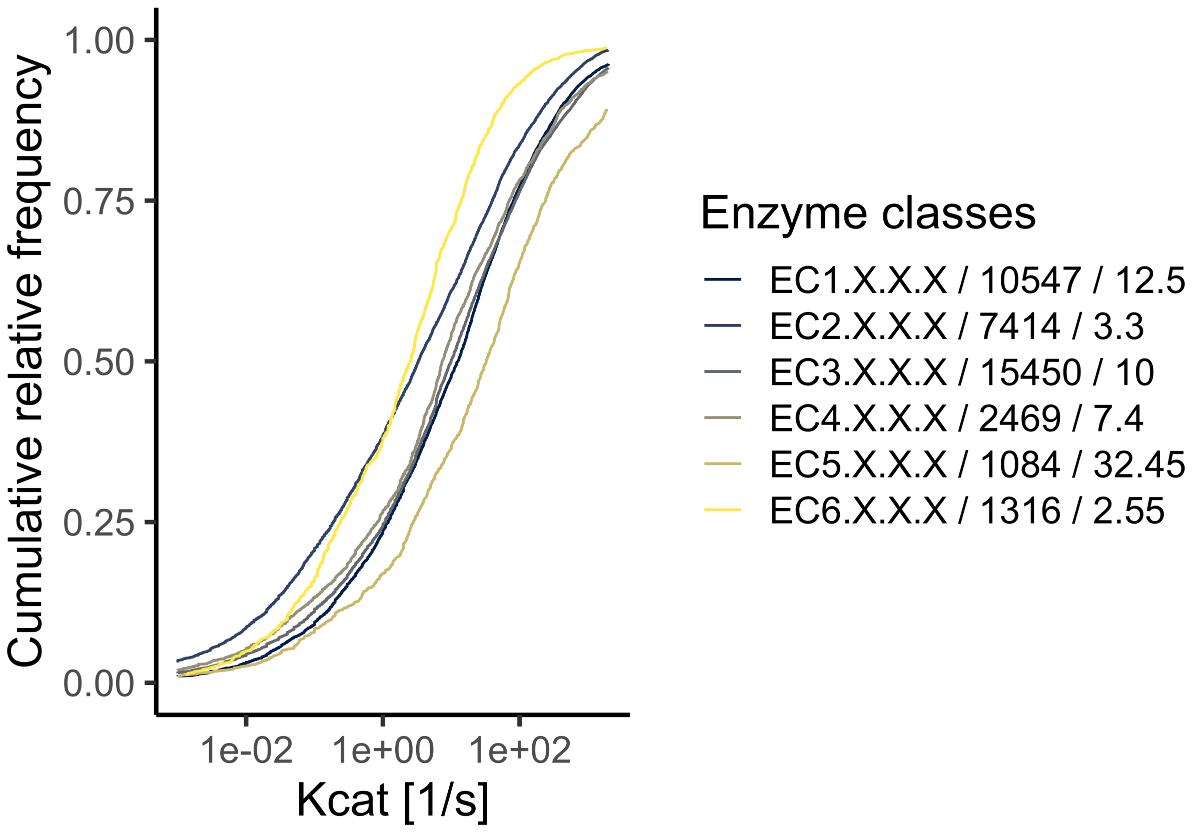

Figure S1.10: Cumulative distributions for Kcat values per enzyme family

In order to test if distributions of k_{cat} values differ significantly amongst enzyme families, a pairwise Kolmogorov-Smirnov statistical test is applied to every possible combination of distributions.

Table S1.5: p-values under the pairwise Kolmogorov-Smirnov two-tailed statistical test

	EC1.X.X.X	EC2.X.X.X	EC3.X.X.X	EC4.X.X.X	EC5.X.X.X	EC6.X.X.X
EC1.X.X.X	1.00e+00	0	0.0000249	0.0000000	0	0
EC2.X.X.X	0.00e+00	1	0.0000000	0.0000000	0	0
EC3.X.X.X	2.49e-05	0	1.0000000	0.0007033	0	0
EC4.X.X.X	0.00e+00	0	0.0007033	1.0000000	0	0
EC5.X.X.X	0.00e+00	0	0.0000000	0.0000000	1	0
EC6.X.X.X	0.00e+00	0	0.0000000	0.0000000	0	1

As expected, enzyme catalytic activity distributions differ significantly across enzyme families. It should be noted that enzyme families represent groups of biochemical reactions wich drastically differ in their mechanisms. To dig deeper into the catalytic specialization of different enzyme sub-families, wild cards can be introduced into the dataset EC numbers to obtain the subfamilies with more data entries available.

Table S1.6: Top10 represented enzyme subfamilies (2 wild-cards)

Enzyme groups	Number of entries
EC3.4.X.X	6292
EC1.1.X.X	4086
EC3.2.X.X	3189
EC3.1.X.X	2980
EC2.7.X.X	2196
EC3.5.X.X	1987
EC2.3.X.X	1467
EC2.4.X.X	1318
EC4.1.X.X	1034
EC1.14.X.X	1005

The top1 represented enzyme subfamily (top1 EC# w/1 wild-card) comprises different groups of enzymes (EC#'s w/2 wild-cards), the next plot shows how different k_{cat} distributions are for these enzyme groups.

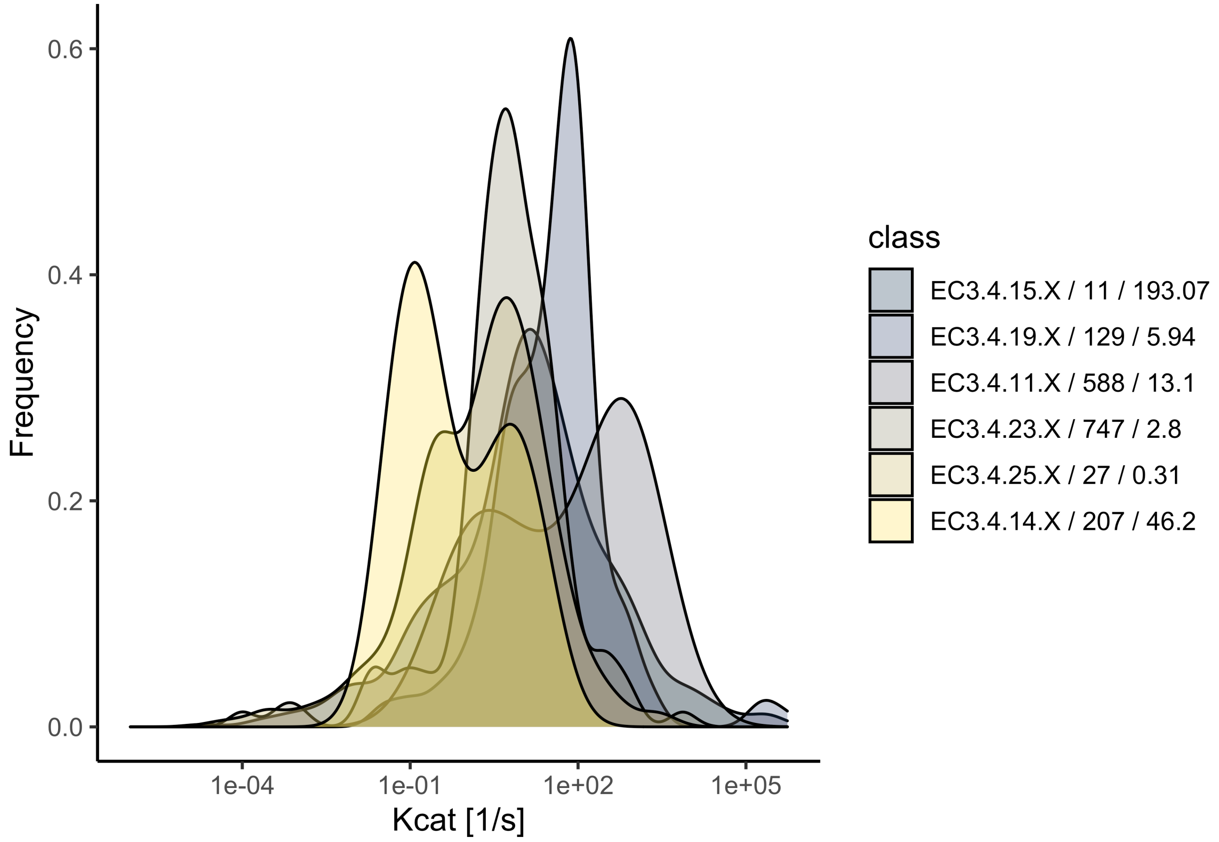

Figure S1.11: Cumulative distributions for Kcat values for different enzyme groups in the most represented enzyme subfamily (2 wild-cards)

Table S1.7: p-values under the pairwise Kolmogorov-Smirnov two-tailed statistical test

	EC3.4.15.X	EC3.4.19.X	EC3.4.11.X	EC3.4.23.X	EC3.4.25.X	EC3.4.14.X
EC3.4.15.X	1.0000000	0.0175175	0.1215817	0.0064116	0.0105730	0.0503262
EC3.4.19.X	0.0175175	1.0000000	0.0000472	0.0000022	0.0000615	0.0000000
EC3.4.11.X	0.1215817	0.0000472	1.0000000	0.0000000	0.0000648	0.0000003
EC3.4.23.X	0.0064116	0.0000022	0.0000000	1.0000000	0.0142993	0.0000000
EC3.4.25.X	0.0105730	0.0000615	0.0000648	0.0142993	1.0000000	0.0000000
EC3.4.14.X	0.0503262	0.0000000	0.0000003	0.0000000	0.0000000	1.0000000

The same can be observed for the top-2 represented enzyme subfamily.

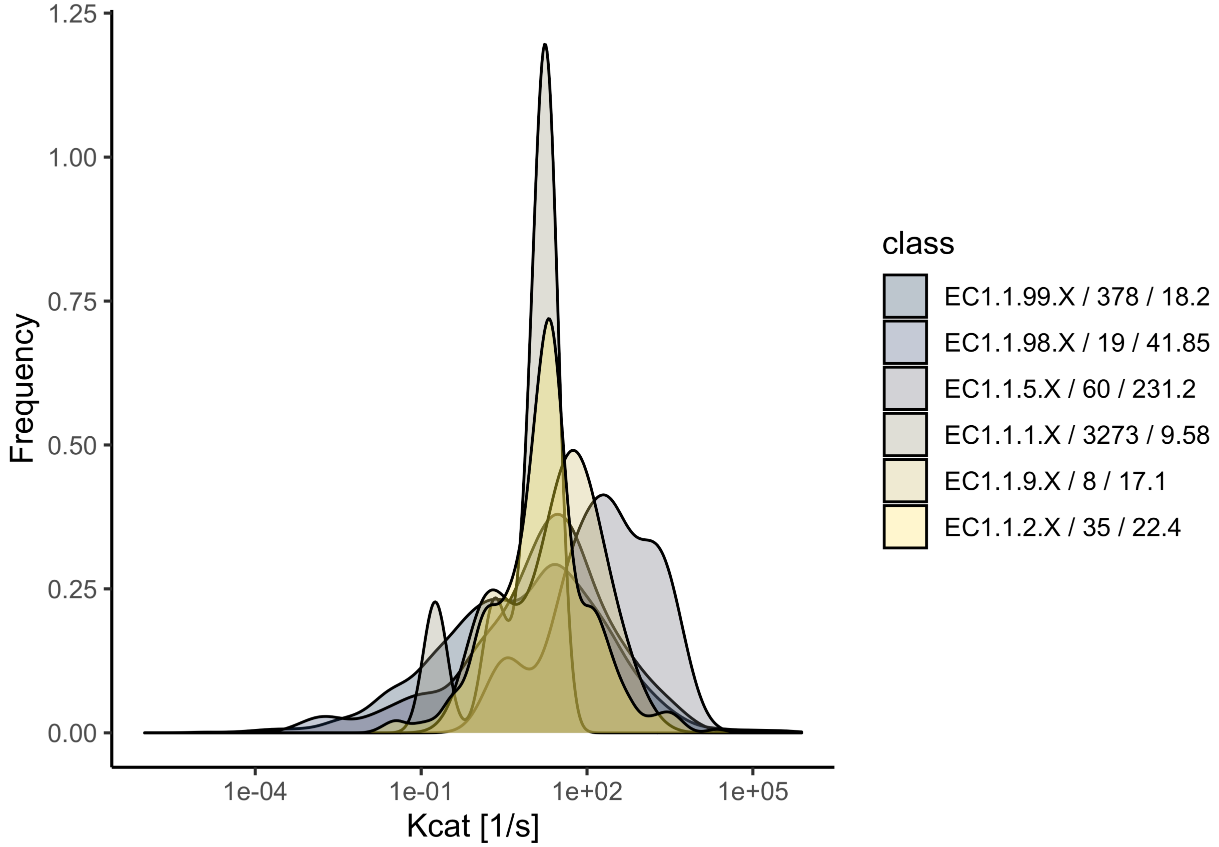

Figure S1.12: Cumulative distributions for Kcat values for different enzyme groups in the most represented enzyme subfamily (2 wild-cards)

Table S1.8: p-values under the pairwise Kolmogorov-Smirnov two-tailed statistical test

	EC1.1.99.X	EC1.1.98.X	EC1.1.5.X	EC1.1.1.X	EC1.1.9.X	EC1.1.2.X
EC1.1.99.X	1.0000000	0.0466039	0.0000000	0.0000000	0.0644177	0.3665778
EC1.1.98.X	0.0466039	1.0000000	0.0017105	0.0724222	0.0224137	0.7081543
EC1.1.5.X	0.0000000	0.0017105	1.0000000	0.0000000	0.0000329	0.0000097
EC1.1.1.X	0.0000000	0.0724222	0.0000000	1.0000000	0.1377232	0.2677261
EC1.1.9.X	0.0644177	0.0224137	0.0000329	0.1377232	1.0000000	0.0638382
EC1.1.2.X	0.3665778	0.7081543	0.0000097	0.2677261	0.0638382	1.0000000

It can be seen that kinetic parameters not only differ significantly across general enzyme families (top-level EC numbers), but also across different enzyme subgroups (EC#'s w/ 2 wild-cards) within these families.

3.3.2 k_{cat} distributions per metabolic context

As all k_{cat} entries were assigned a metabolic subgroup, according to the KEGG pathways classification, the effect of metabolic context on k_{cat} values differentiation was also assessed. $Fig.S1.13$ shows that, in general, enzymes in the central carbon and energy metabolism pathways tend to be significantly faster than those involved in amino acids, lipids and secondary metabolism.

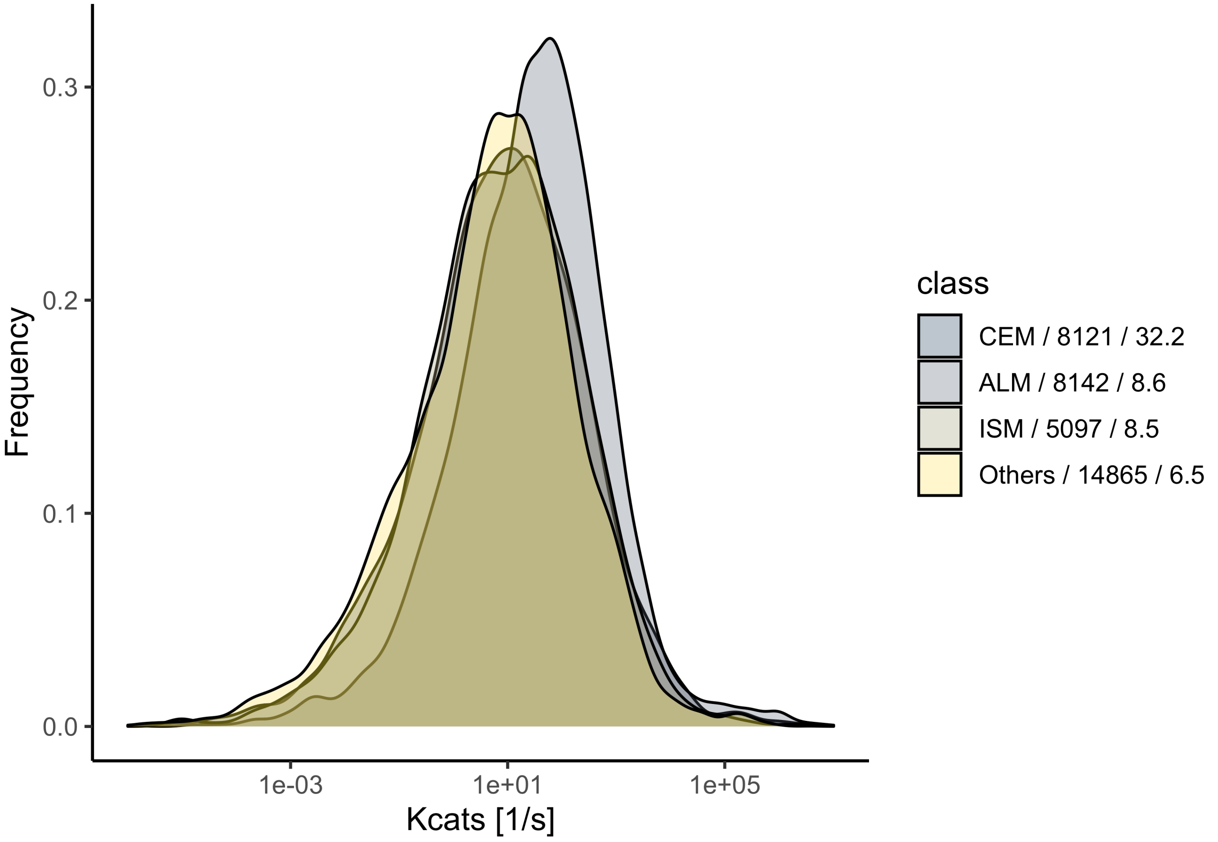

Figure S1.13: Kcat distributions sorted by metabolic context of enzymes

3.3.3 k_{cat} distributions per organism Kingdoms

Each entry of the retrieved k_{cat} values contains information regarding substrate and organism, it is then possible to assign a phylogenetic classification to each entry by retrieving the classification of the organism of origin from the KEGG organisms database. Therefore, comparison of k_{cat} distributions by different taxonomy levels is possible. $Fig.S1.14$ shows k_{cat} cumulative distributions when sorted by organism kingdom, where it is evident that activity values for enzymes in microbial organisms (fungi, bacteria and archaea) tend to be significantly higher than those for other organisms such as animals and plants.

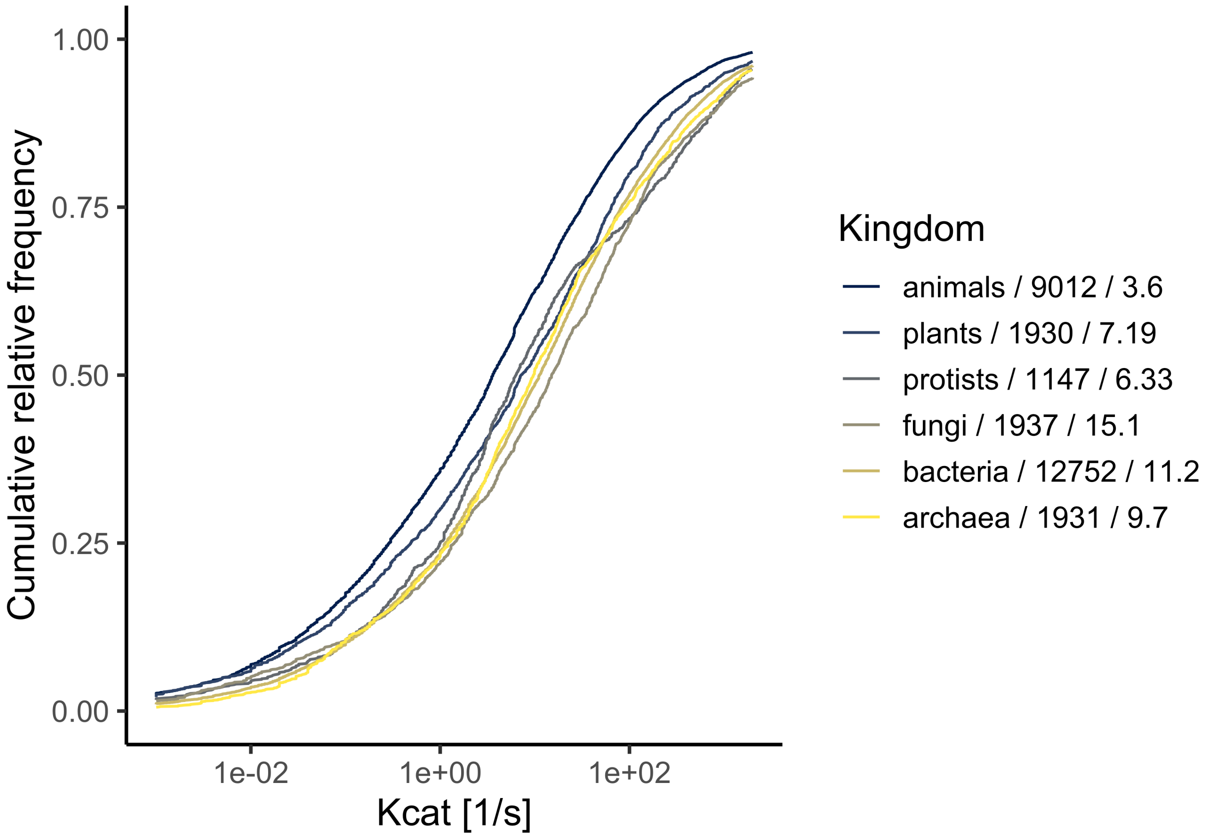

Figure S1.14: Cumulative distributions for Kcat values per organism kingdoms

Table S1.9: p-values under the pairwise Kolmogorov-Smirnov two-tailed statistical test

	animals	plants	protists	fungi	bacteria	archaea
animals	1	0.00e+00	0.0000000	0.00e+00	0.0000000	0.0000000
plants	0	1.00e+00	0.0000510	4.00e-07	0.0000004	0.0000479
protists	0	5.10e-05	1.0000000	1.00e-07	0.0000606	0.0129826
fungi	0	4.00e-07	0.0000001	1.00e+00	0.0000341	0.0000252
bacteria	0	4.00e-07	0.0000606	3.41e-05	1.0000000	0.1634046
archaea	0	4.79e-05	0.0129826	2.52e-05	0.1634046	1.0000000

However, due to the impact of metabolic context on k_{cat} values differentiation ($Fig.S1.13$), this should also be taken into account when studying the role of phylogeny. $Figs.S1.15-20$ show that enzymes in Fungi organisms tend to be significantly faster than those for other organisms across **all** metabolic contexts.

Additionally, it was found that enzymes for higher organisms, such as animals, tend to display activity values that are lower, in general, than those for microbial organisms across **all** studied metabolic groups.

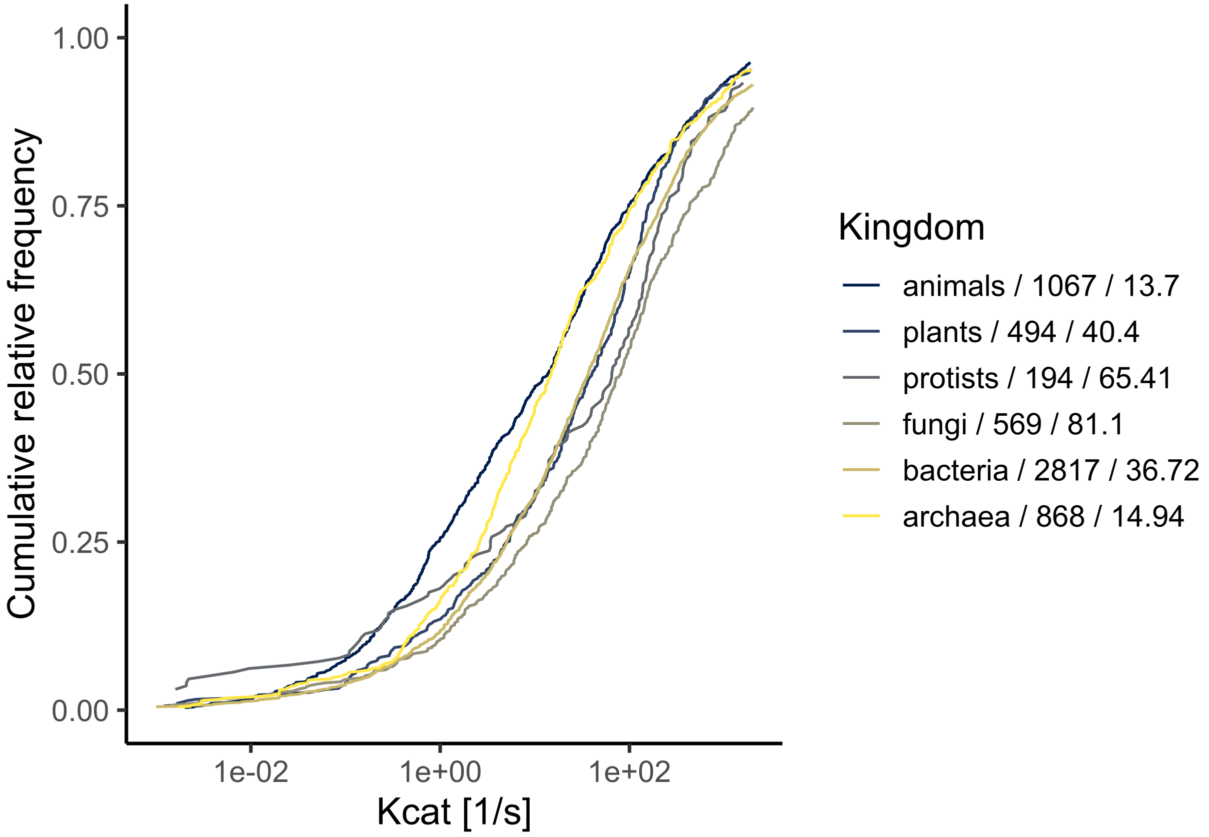

Figure S1.15: Cumulative distributions for Kcat values in CEM per organism kingdoms

Table S1.10: p-values under the pairwise Kolmogorov-Smirnov two-tailed statistical test

	animals	plants	protists	fungi	bacteria	archaea
animals	1.00e+00	0.0000000	0.0000001	0.0000000	0.0000000	3.41e-05
plants	0.00e+00	1.0000000	0.1084608	0.0000540	0.2147233	1.00e-07
protists	1.00e-07	0.1084608	1.0000000	0.1037212	0.0420287	1.80e-06
fungi	0.00e+00	0.0000540	0.1037212	1.0000000	0.0000002	0.00e+00
bacteria	0.00e+00	0.2147233	0.0420287	0.0000002	1.0000000	0.00e+00
archaea	3.41e-05	0.0000001	0.0000018	0.0000000	0.0000000	1.00e+00

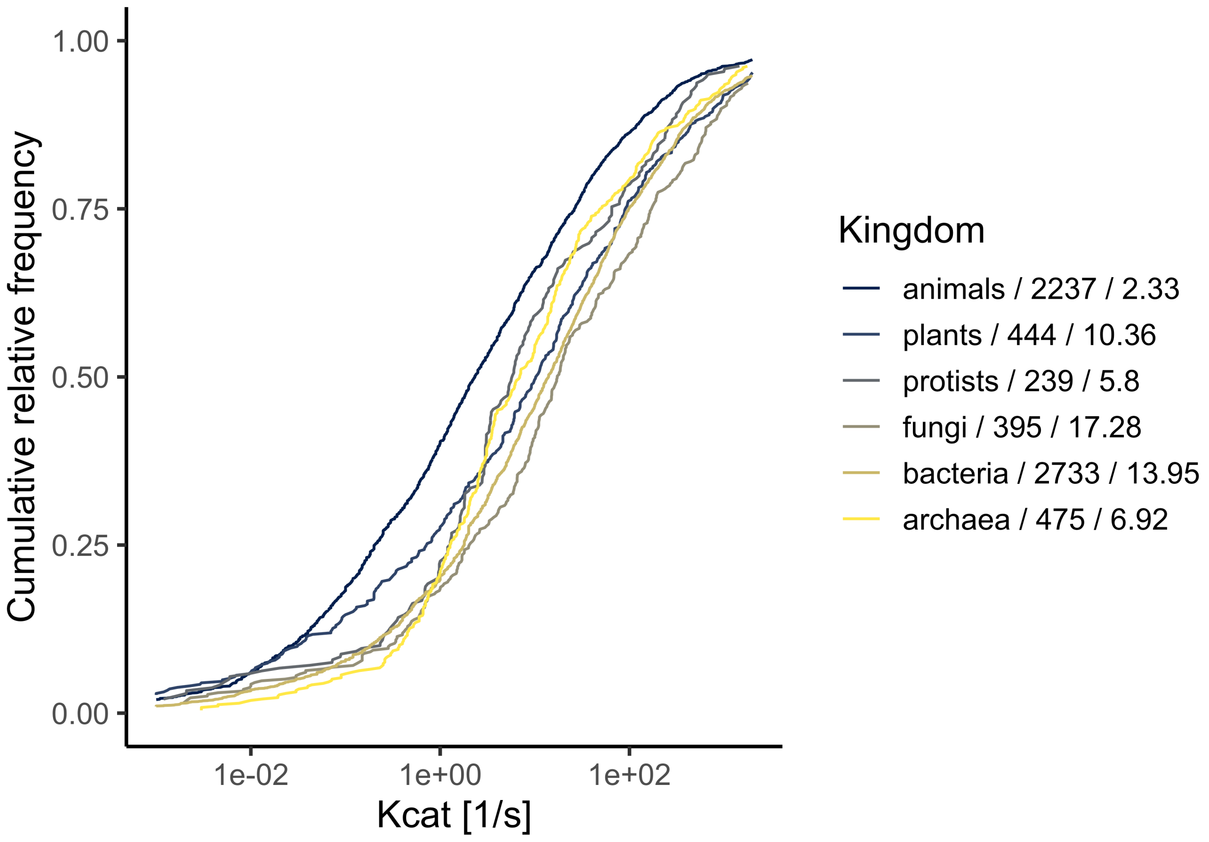

Figure S1.16: Cumulative distributions for Kcat values in ALM per organism kingdoms

Table S1.11: p-values under the pairwise Kolmogorov-Smirnov two-tailed statistical test

	animals	plants	protists	fungi	bacteria	archaea
animals	1e+00	0.0000000	0.0000001	0.0000000	0.0000000	0.0000000
plants	0e+00	1.0000000	0.0795835	0.0110713	0.0094757	0.0012962
protists	1e-07	0.0795835	1.0000000	0.0000212	0.0005014	0.6270489
fungi	0e+00	0.0110713	0.0000212	1.0000000	0.0476123	0.0000556
bacteria	0e+00	0.0094757	0.0005014	0.0476123	1.0000000	0.0000591
archaea	0e+00	0.0012962	0.6270489	0.0000556	0.0000591	1.0000000

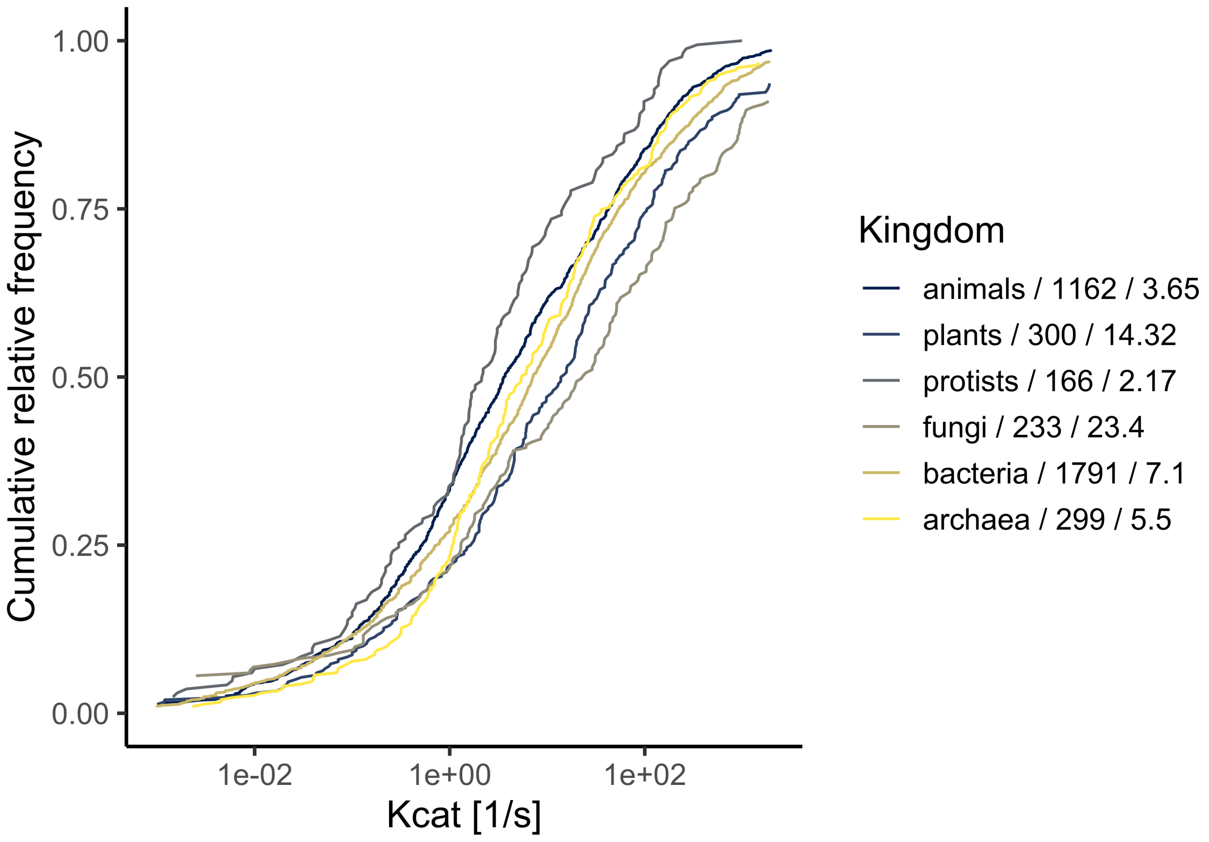

Figure S1.17: Cumulative distributions for Kcat values in ISM per organism kingdoms

Table S1.12: p-values under the pairwise Kolmogorov-Smirnov two-tailed statistical test

	animals	plants	protists	fungi	bacteria	archaea
animals	1.0000000	0.0000038	0.0420024	0.0000005	0.0000303	0.0102206
plants	0.0000038	1.0000000	0.0000009	0.1433978	0.0197837	0.0138157
protists	0.0420024	0.0000009	1.0000000	0.0000000	0.0000186	0.0049434
fungi	0.0000005	0.1433978	0.0000000	1.0000000	0.0000094	0.0000057
bacteria	0.0000303	0.0197837	0.0000186	0.0000094	1.0000000	0.1498901
archaea	0.0102206	0.0138157	0.0049434	0.0000057	0.1498901	1.0000000

3.3.4 k_{cat} distributions per metabolic pathways groups per organism Kingdoms

It has been shown that, overall, enzymes in central carbon and energy metabolism have been reported to have higher activity values than those in other metabolic contexts ($Fig.S1.13$)). Nonetheless, enzyme activity also seems to be related to the phylogeny of their organism of origin. In order to assess if CEM related enzymes are faster than others, distributions of k_cat values for each studied metabolic context were statistically compared for each of the KEGG kingdoms of life.

$Figs.S1.18 - 23$ show cumulative distributions for enzymes sorted by different metabolic groups for each of the KEGG kingdoms of life. Notably, central carbon and energy metabolism enzymes present higher values, on average, than those in other metabolic contexts. Notably, when focusing on enzymes for Fungi

and Bacteria organisms (two well-studied phylogenetic groups) it turns out that all k_cat value distributions for the studied metabolic contexts, differ significantly ($Tables S1.16 - 17$).

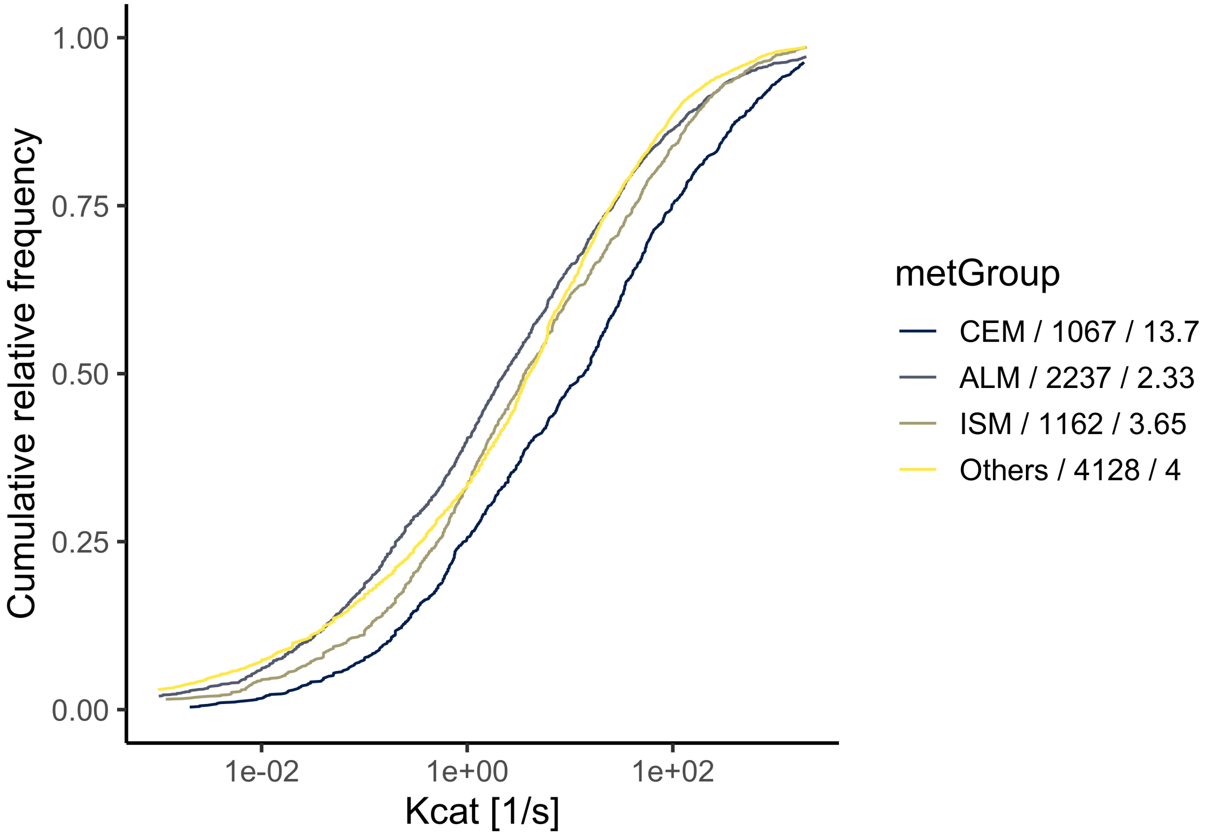

Figure S1.18: Cumulative distributions for Kcat values by metabolic subgroup for Animals

Table S1.13: p-values under the pairwise Kolmogorov-Smirnov two-tailed statistical test

	CEM	ALM	ISM	Others
CEM	1	0.00e+00	0.0000000	0.0000000
ALM	0	1.00e+00	0.0000194	0.0000000
ISM	0	1.94e-05	1.0000000	0.0021873
Others	0	0.00e+00	0.0021873	1.0000000

Animals

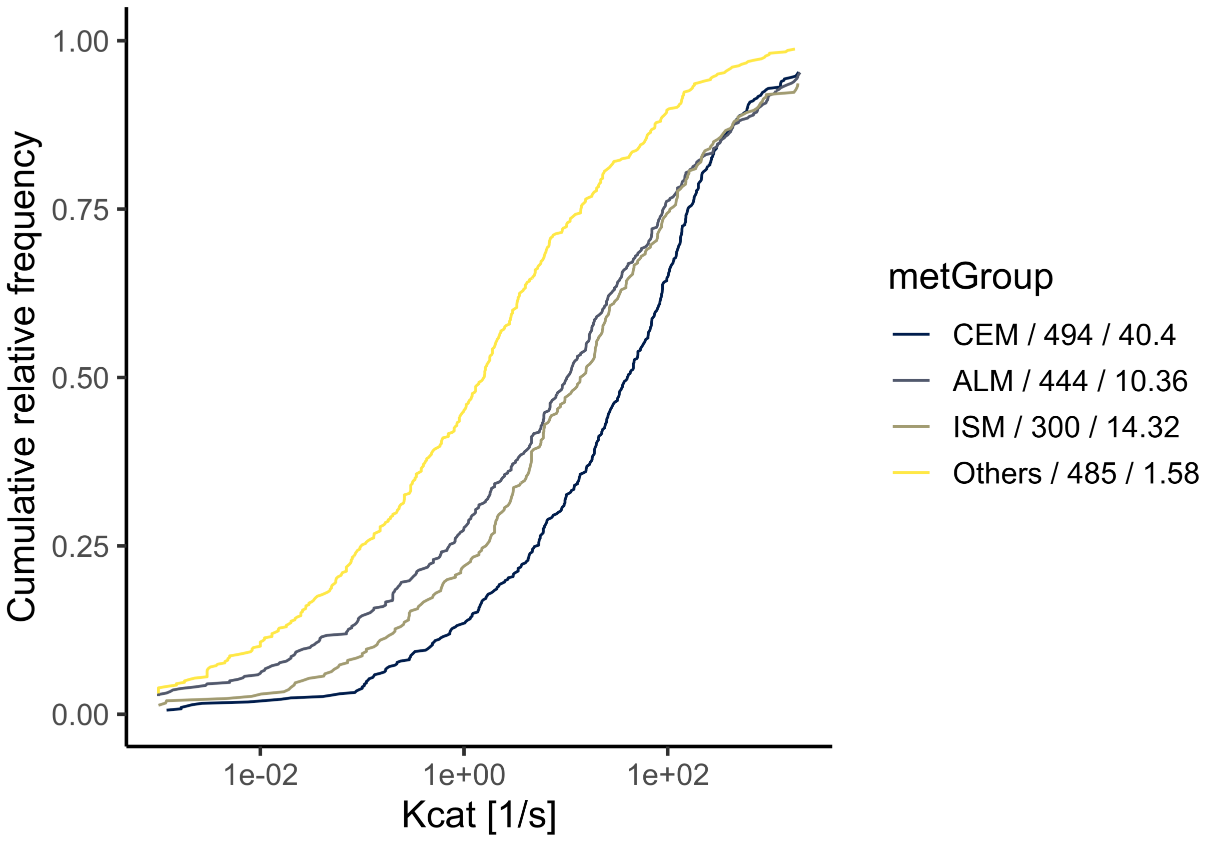

Figure S1.19: Cumulative distributions for Kcat values by metabolic subgroup for Plants

Table S1.14: p-values under the pairwise Kolmogorov-Smirnov two-tailed statistical test

	CEM	ALM	ISM	Others
CEM	1.0000000	0.000000	0.0001715	0
ALM	0.0000000	1.000000	0.2961840	0
ISM	0.0001715	0.296184	1.0000000	0
Others	0.0000000	0.000000	0.0000000	1

Plants

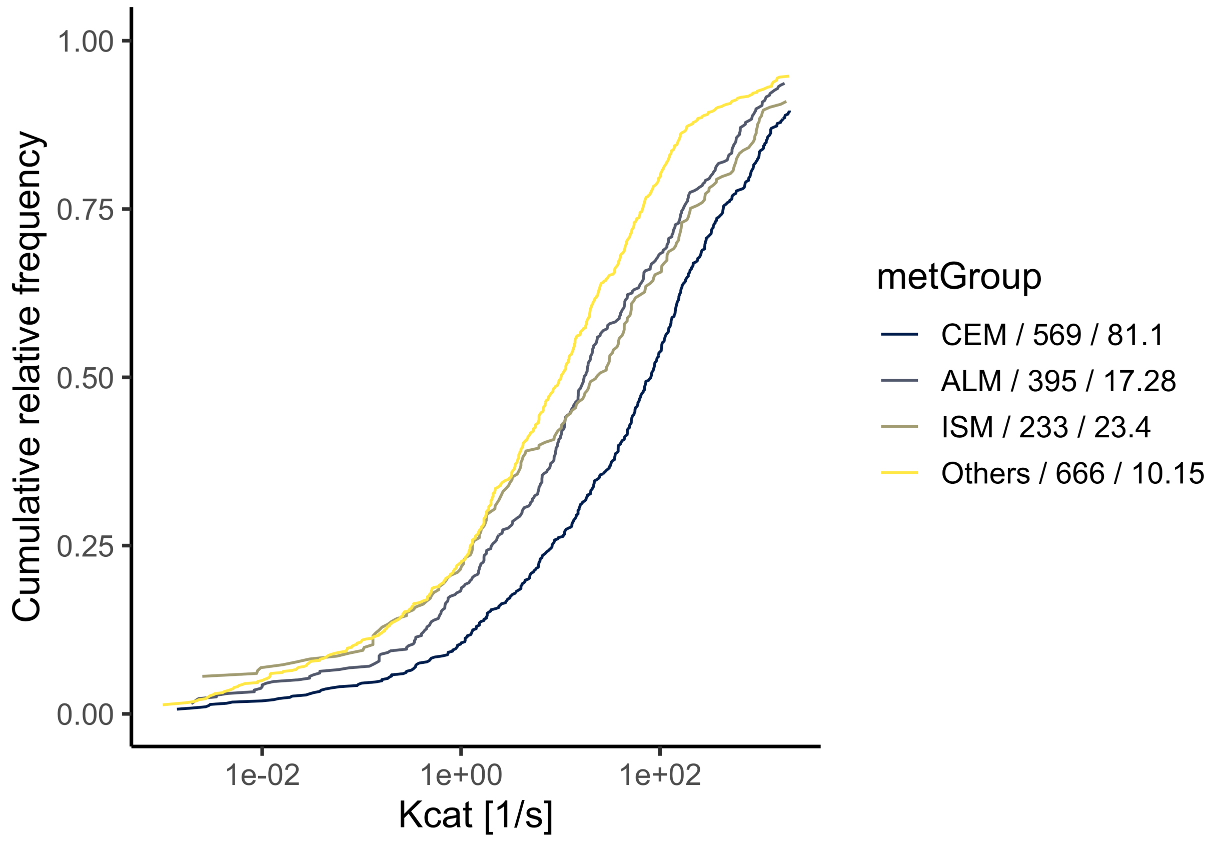

Figure S1.20: Cumulative distributions for Kcat values by metabolic subgroup for Fungi

Table S1.15: p-values under the pairwise Kolmogorov-Smirnov two-tailed statistical test

	CEM	ALM	ISM	Others
CEM	1.00e+00	0.0000000	0.0000102	0.0000000
ALM	0.00e+00	1.0000000	0.2495139	0.0004581
ISM	1.02e-05	0.2495139	1.0000000	0.0006279
Others	0.00e+00	0.0004581	0.0006279	1.0000000

Fungi

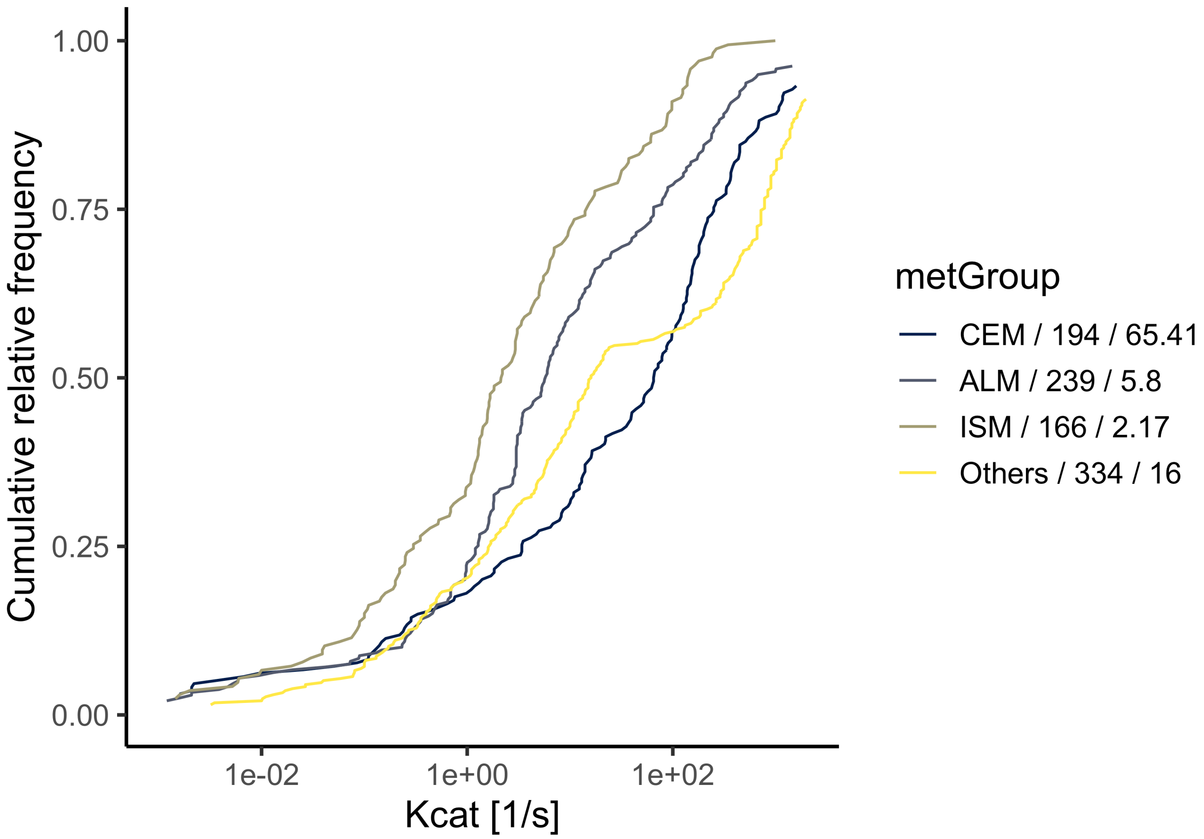

Figure S1.21: Cumulative distributions for Kcat values by metabolic subgroup for Protists

Table S1.16: p-values under the pairwise Kolmogorov-Smirnov two-tailed statistical test

	CEM	ALM	ISM	Others
CEM	1.0000000	0.0000001	0.0000000	0.0014397
ALM	0.0000001	1.0000000	0.0016707	0.0000000
ISM	0.0000000	0.0016707	1.0000000	0.0000000
Others	0.0014397	0.0000000	0.0000000	1.0000000

Protists

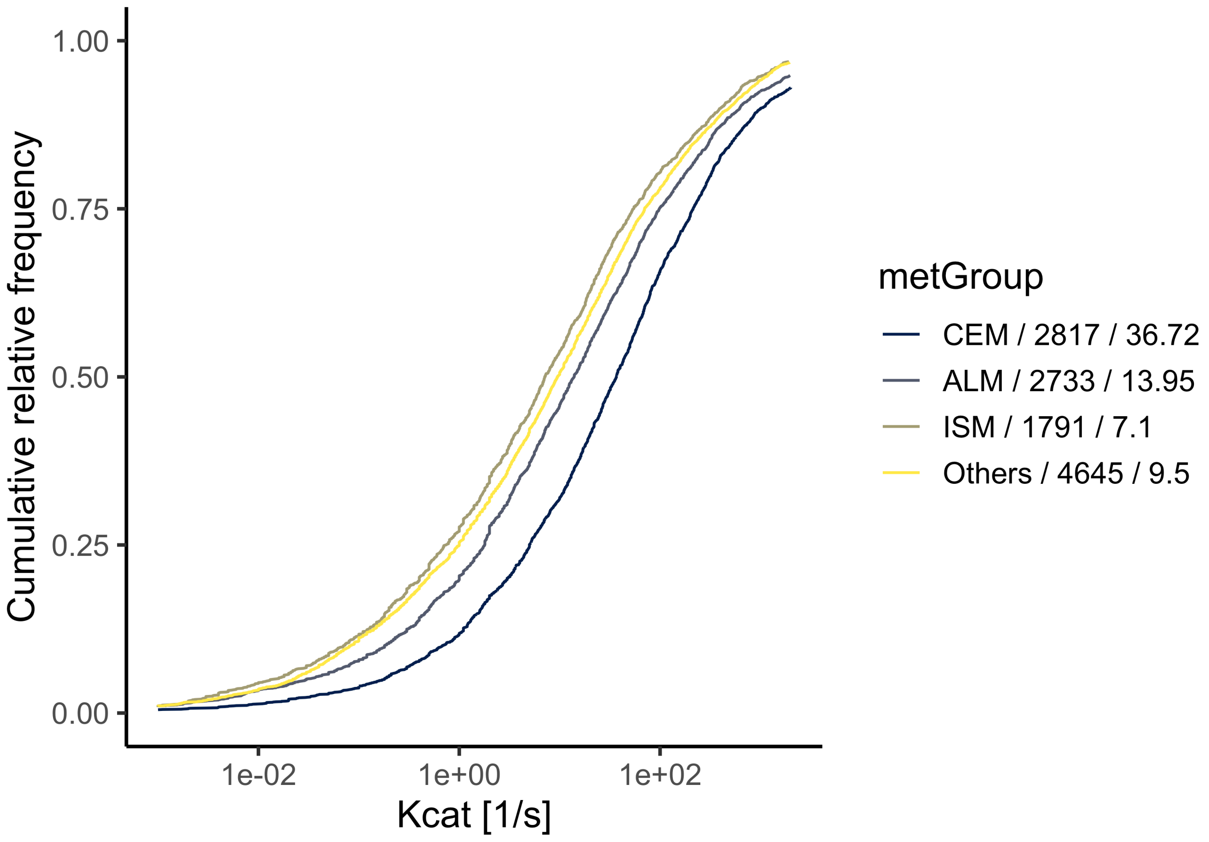

Figure S1.22: Cumulative distributions for Kcat values by metabolic subgroup for Bacteria

Table S1.17: p-values under the pairwise Kolmogorov-Smirnov two-tailed statistical test

	CEM	ALM	ISM	Others
CEM	1	0.00e+00	0.0000000	0.0000000
ALM	0	1.00e+00	0.0000004	0.0000188
ISM	0	4.00e-07	1.0000000	0.0280320
Others	0	1.88e-05	0.0280320	1.0000000

Bacteria

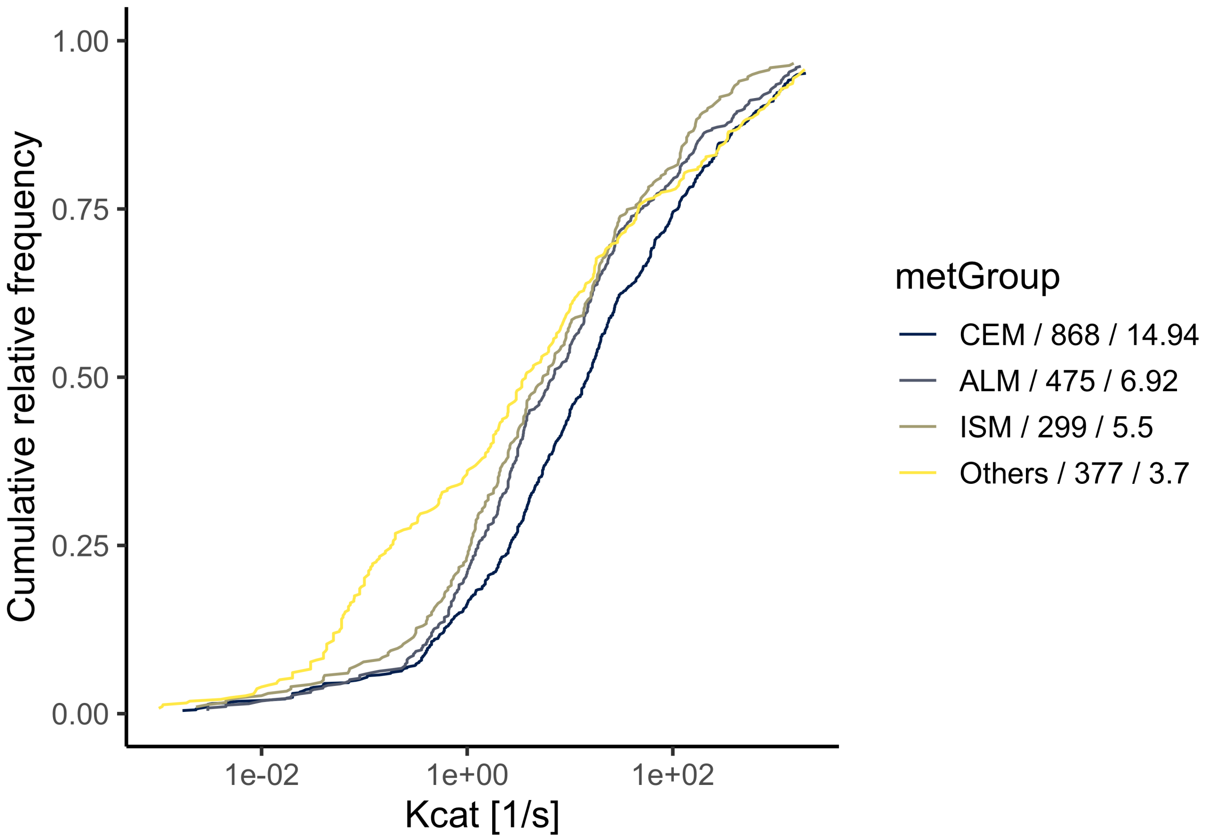

Figure S1.23: Cumulative distributions for Kcat values by metabolic subgroup for Archaea

Table S1.18: p-values under the pairwise Kolmogorov-Smirnov two-tailed statistical test

	CEM	ALM	ISM	Others
CEM	1.00e+00	0.0000211	0.0000333	0e+00
ALM	2.11e-05	1.0000000	0.8120868	0e+00
ISM	3.33e-05	0.8120868	1.0000000	8e-05
Others	0.00e+00	0.0000000	0.0000800	1e+00

Archaea
