# Supplementary material for: Reconstruction of a catalogue of genome-scale metabolic models with enzymatic constraints using GECKO 2.0
Source: Nat Commun. 2022 Jun 30;13:3766. doi: 10.1038/s41467-022-31421-1 (PMC9246944; doi:10.1038/s41467-022-31421-1)
Supplement: Supplementary file 2 — reporting summary [file 41467_2022_31421_MOESM2_ESM.pdf]

nature research

Corresponding author(s): Profr. Jens N ielsen

Last updated by author(s): June 10, 2022



# Reporting Summary

Nature Research wishes to improve the reproducibility of the work that we publish. This form provides structure for consistency and transparency in reporting. For further information on Nature Research policies, see our Editorial Policies and the Editorial Policy Checklist.

## Statistics

For all statistical analyses, confirm that the following items are present in the figure legend, table legend, main text, or Methods section.

| n/a | Confirmed | |
|---|---|---|
| ☐ | ☒ | The exact sample size ($n$) for each experimental group/condition, given as a discrete number and unit of measurement |
| ☐ | ☒ | A statement on whether measurements were taken from distinct samples or whether the same sample was measured repeatedly |
| ☐ | ☒ | The statistical test(s) used AND whether they are one- or two-sided<br>*Only common tests should be described solely by name; describe more complex techniques in the Methods section.* |
| ☐ | ☒ | A description of all covariates tested |
| ☐ | ☒ | A description of any assumptions or corrections, such as tests of normality and adjustment for multiple comparisons |
| ☐ | ☒ | A full description of the statistical parameters including central tendency (e.g. means) or other basic estimates (e.g. regression coefficient) AND variation (e.g. standard deviation) or associated estimates of uncertainty (e.g. confidence intervals) |
| ☐ | ☒ | For null hypothesis testing, the test statistic (e.g. $F$, $t$, $r$) with confidence intervals, effect sizes, degrees of freedom and $P$ value noted<br>*Give P values as exact values whenever suitable.* |
| ☒ | ☐ | For Bayesian analysis, information on the choice of priors and Markov chain Monte Carlo settings |
| ☒ | ☐ | For hierarchical and complex designs, identification of the appropriate level for tests and full reporting of outcomes |
| ☒ | ☐ | Estimates of effect sizes (e.g. Cohen's $d$, Pearson's $r$), indicating how they were calculated |

*Our web collection on statistics for biologists contains articles on many of the points above.*

## Software and code

Policy information about availability of computer code

| | |
|---|---|
| Data collection | The GECKO toolbox V 2.0 contains a python module (https://github.com/SysBioChalmers/GECKO/tree/master/geckomat/brenda__parser), which main functionality is to download all the kinetic parameters that are necessary from the BRENDA database (https://www.brenda-enzymes.org/). This parameter retrieval is performed every 6 months in order to update the toolbox. |
| Data analysis | Numerical results and analyses in this manuscript were obtained using custom code wrtitten in MATLAB R 2018B and R version 3.3.3, available at: https://github.com/SysBioChalmers/GECKO2__simulations/releases/tag/v 1.0.1. Flux balance analysis simulations were ran with The RAVEN toolbox v 2.4.3. All the generated computational models can be found at https://github.com/SysBioChalmers/ecModels/releases/tag/v 1.0.0. All the necessary scripts for reproducing the kcat parameters analysis in the Supplementary Information File 1 are available at: https://github.com/SysBioChalmers/Enzyme-parameters-analysis/releases/tag/v 1.0.0. |

For manuscripts utilizing custom algorithms or software that are central to the research but not yet described in published literature, software must be made available to editors and reviewers. We strongly encourage code deposition in a community repository (e.g. GitHub). See the Nature Research guidelines for submitting code & software for further information.

## Data

Policy information about availability of data

All manuscripts must include a data availability statement. This statement should provide the following information, where applicable:

- Accession codes, unique identifiers, or web links for publicly available datasets
- A list of figures that have associated raw data
- A description of any restrictions on data availability

- Data supporting the findings of this work are available within the paper and its Supplementary files.
- A reporting summary for this Article is available as a Supplementary Information file.

- Mass spectrometry raw data that support the findings of this study have been deposited in PRIDE database74 with the dataset identifier PXD012836. The processed proteomics datasets are available in our GitHub repository at: https://github.com/SysBioChalmers/GECKO2_simulations/tree/v1.0.1/data/proteomics. All collected kinetic data for the study presented in Supplementary Information File are available at: https://github.com/SysBioChalmers/Enzyme-parameters-analysis/tree/master/data. The generated computational models used for this study are available at: https://github.com/SysBioChalmers/ecModels/tree/v1.0.0. Data for reproduction of all main and supplementary figures are provided in Data Source file 1, Data Source File 2 and Data Source File 3.

# Field-specific reporting

Please select the one below that is the best fit for your research. If you are not sure, read the appropriate sections before making your selection.

[x] Life sciences [ ] Behavioural & social sciences [ ] Ecological, evolutionary & environmental sciences

For a reference copy of the document with all sections, see nature.com/documents/nr-reporting-summary-flat.pdf

# Life sciences study design

All studies must disclose on these points even when the disclosure is negative.

| | |
|---|---|
| Sample size | For protein abundance quantification no calculation was performed to determine sample size. Sample sizes were chosen to cover the maximum number of species and stress conditions while maintaining just enough replicates to inform statistical analysis of biological variability. |
| Data exclusions | No samples were excluded for protein abundance quantification. |
| Replication | Each experimental condition included three biological replicates, all successful, taken from separate fermenters. After initial processing of the protein abundance data, proteins that were measured with a relative standard deviation higher than one across triplicates (for a given condition) were discarded. |
| Randomization | No randomization of samples allocation was performed in this study. All analyzed samples (for protein quantification) were obtained from biological samples for three different yeast species subjected to the same environmental stress conditions. |
| Blinding | Blinding was not employed for this study as it was necessary to know the species from which samples were obtained in order to perform the protein quantification analysis. |

# Reporting for specific materials, systems and methods

We require information from authors about some types of materials, experimental systems and methods used in many studies. Here, indicate whether each material, system or method listed is relevant to your study. If you are not sure if a list item applies to your research, read the appropriate section before selecting a response.

## Materials & experimental systems

| n/a | Involved in the study | |
|---|---|---|
| [x] | [ ] | Antibodies |
| [x] | [ ] | Eukaryotic cell lines |
| [x] | [ ] | Palaeontology and archaeology |
| [x] | [ ] | Animals and other organisms |
| [x] | [ ] | Human research participants |
| [x] | [ ] | Clinical data |
| [x] | [ ] | Dual use research of concern |

## Methods

| n/a | Involved in the study | |
|---|---|---|
| [x] | [ ] | ChIP-seq |
| [x] | [ ] | Flow cytometry |
| [x] | [ ] | MRI-based neuroimaging |
